# Chronology of critical events in neonatal rat ventricular myocytes occurring during reperfusion after simulated ischemia

**DOI:** 10.1371/journal.pone.0212076

**Published:** 2019-02-07

**Authors:** Katie J. Sciuto, Steven W. Deng, Alonso Moreno, Alexey V. Zaitsev

**Affiliations:** 1 Nora Eccles Harrison Cardiovascular Research and Training Institute, University of Utah, Salt Lake City, Utah, United States of America; 2 Department of Bioengineering, University of Utah, Salt Lake City, Utah, United States of America; 3 Department of Internal Medicine, School of Medicine, University of Utah, Salt Lake City, Utah, United States of America; Rush University Medical Center, UNITED STATES

## Abstract

While an ischemic insult poses a lethal danger to myocardial cells, a significant proportion of cardiac myocytes remain viable throughout the ischemic episode and die, paradoxically, only after the blood flow is reinstated. Despite decades of research, the actual chronology of critical events leading to cardiomyocyte death during the reperfusion phase remains poorly understood. Arguably, identification of the pivotal event in this setting is necessary to design effective strategies aimed at salvaging the myocardium after an ischemic attack. Here we used neonatal rat ventricular myocytes (NRVMs) subjected to 20–30 min of simulated ischemia followed by 1 hour of “reperfusion”. Using different combinations of spectrally-compatible fluorescent indicators, we analyzed the relative timing of the following events: (1) abnormal increase in cytoplasmic [Ca^2+^] (T_CaCy_); (2) abnormal increase in mitochondrial [Ca^2+^] (T_CaMi_); (3) loss of mitochondrial inner membrane potential (ΔΨ_m_) indicating mitochondrial permeability transitions (T_MPT_); (4) sacrolemmal permeabilization (SP) to the normally impermeable small fluorophore TO-PRO3 (T_SP_). In additional experiments we also analyzed the timing of abnormal uptake of Zn^2+^ into the cytoplasm (T_ZnCy_) relative to T_CaCy_ and T_SP_. We focused on those NRVMs which survived anoxia, as evidenced by at least 50% recovery of ΔΨ_m_ and the absence of detectable SP. In these cells, we found a consistent sequence of critical events in the order, from first to last, of T_CaCy_, T_CaMi,_ T_MPT_, T_SP_. After detecting T_CaCy_ and T_CaMi_, abrupt switches between 1.1 mM and nominally zero [Ca^2+^] in the perfusate quickly propagated to the cytoplasmic and mitochondrial [Ca^2+^]. Depletion of the sarcoplasmic reticulum with ryanodine (5 μM)/thapsigargin (1 μM) accelerated all events without changing their order. In the presence of ZnCl_2_ (10–30 μM) in the perfusate we found a consistent timing sequence T_CaCy_ < T_Zn_ ≤ T_SP_. In some cells ZnCl_2_ interfered with Ca^2+^ uptake, causing “steps” or “gaps” in the [Ca^2+^]_Cy_ curve, a phenomenon never observed in the absence of ZnCl_2_. Together, these findings suggest an evolving permeabilization of NRVM’s sarcolemma during reoxygenation, in which the expansion of the pore size determines the timing of critical events, including T_MPT._

## Introduction

Cardiac disease remains the leading cause of death in developed countries, and worldwide. Central to cardiac disease is myocardial ischemia/reperfusion (I/R). Whereas the early restoration of blood flow and oxygen delivery to the affected region of the heart is the best remedy to limit myocardial infarct, it is now well established that a significant proportion of cardiac myocytes survive ischemic episode and die specifically during reperfusion. These myocytes are potentially salvageable by interventions applied during reperfusion which can prevent or disrupt the cascade of cellular events leading to irreversible injury. Whereas decades of prior research have provided very detailed information regarding interconnected ionic, metabolic, and functional alterations in the course of myocardial I/R, there is still a poor understanding of how any, or all, of these changes lead to catastrophic cellular events, determining the fate of individual myocytes, or the entire organ. Previous studies point to three major pathophysiological factors, which can explain the catastrophic transitions in the context of myocardial I/R. These include a shutdown of mitochondrial inner membrane potential (ΔΨ_m_) resulting from an abrupt increase in mitochondrial membrane leak; plasma membrane (sarcolemmal) permeabilization (SP); and excessive accumulation of Ca^2+^ (Ca^2+^ overload) in the cytoplasm, or sarcoplasmic reticulum, or mitochondrial matrix. In different prior studies one can find almost all possible permutations between these factors in terms of their temporal sequence and cause-effect relationship, depending on the stated hypothesis [1–4]. As a result, the question of the chronology of critical events during reperfusion remains open.

The current prevailing theory puts forward the mitochondrial permeability transition pore opening (MPT) as the pivotal event of the death pathway [5], positioning SP downstream of MPT. Presumably, MPT requires a critical increase in cellular Ca^2+^, but it is not established what causes that increase and when it occurs. Also, the pathway from MPT to SP remains obscure [6]. A recent study from our lab found that in a whole rabbit heart model, SP and MPT are virtually simultaneous, but inhibition of the MPT pore by Cyclosporine A (CsA) creates a detectable separation between events, such that SP occurs ahead of MPT [7]. We speculated that SP is the primary event in cardiomyocyte death, and provides the source of excessive Ca^2+^ influx, leading to mitochondrial Ca^2+^ concentrations at which MPT is unavoidable even in the presence of CsA. In this case MPT is an epi-phenomenon and perhaps not the best therapeutic target to improve outcomes of myocardial I/R. Indeed, the latest clinical trials of CsA in patients undergoing percutaneous coronary intervention procedure showed lack of benefit [8].

Our study mentioned above [7] lacked information about the dynamics of either cytoplasmic or intra-mitochondrial Ca^2+^ in cells undergoing critical transitions. Another relevant study conducted in the whole mouse heart by Davidson et al. [9] monitored cytoplasmic Ca^2+^ and revealed very slow and long-standing Ca^2+^ waves followed by MPT in a subset of cells exhibiting Ca^2+^ waves. No information about mitochondrial [Ca^2+^] was available. In that study, SP monitored by cellular uptake of calcein occurred “minutes” after MPT. The authors could not explain the source of Ca^2+^ waves preceding MPT, but speculated that those could be due to a “canonical” mechanism of spontaneous Ca^2+^ release from the sarcoplasmic reticulum.

The aim of our study was to ascertain, in an unbiased manner, the chronology of critical cellular events, including cytoplasmic Ca^2+^ overload, mitochondrial Ca^2+^ overload, MPT and SP by simultaneous monitoring of multiple (3 or 4) fluorescent reporters in individual myocytes during I/R. We used NRVM monolayers transfected with a gene encoding for a mitochondrial Ca^2+^ indicator LAR-GECO1.2 [10] and loaded with conventional indicators for cytoplasmic Ca^2+^, ΔΨ_m_, and SP. Our results signify a highly consistent chronology of critical events beginning with cytoplasmic Ca^2+^ overload, followed by mitochondrial Ca^2+^ overload, followed by MPT, followed by ‘canonical’ SP as indicated by cellular uptake of the normally cell-impermeable nucleic acid probe TO-PRO3. Through the process of eliminating potential Ca^2+^ sources and analysis of cellular Zn^2+^ uptake we posit that the initial cytoplasmic Ca^2+^ overload occurs through a non-selective channel/pore whose size and/or permeability expands with time of reperfusion, only eventually permitting passage of ‘canonical’ SP indicators such as TO-PRO3. We discuss the possibility that variations in the putative channel/pore evolution can explain the variability in the timing of critical events observed in different studies [7, 9], or even in different myocytes from the same tissue or monolayer.

## Materials and methods

### Animal ethics statement

The study conformed to the National Institute of Health *Guide for the Care and Use of Laboratory Animals* (8th Edition, 2011) and was approved by the Institutional Animal Care and Use Committee of the University of Utah (Protocol number 17–08005). Animal euthanasia was performed using inhalant isoflurane.

### NRVM preparation and adenoviral infection

Coverslips (no. 0) were manually cut and placed in 48 well tissue culture plates (Genesee Scientific, 25–108) followed by sterilization in 70% ethanol overnight. The next day, the culture plate containing coverslips was thoroughly washed with sterile water before treating for 2 hours at room temperature with 50 μg/mL fibronectin (Sigma F1141-5mg) diluted in PBS. The fibronectin solution was removed and coverslips were allowed to dry for an additional 2 hours before cell plating.

Neonatal rat ventricular myocyte monolayers (NRVMs) were isolated and plated using reagents and a modified protocol from Worthington Biochemical (Worthington, LK003300) (http://worthington-biochem.com/NCIS/default.html). Briefly, hearts from 0–1 day old Sprague Dawley SAS400 (Charles River) rat pups were excised and washed. The ventricles were separated from the rest of the heart and used for the remainder of the protocol. The tissue was minced into fragments approximately < 1 mm^3^, and then incubated in trypsin solution overnight at 4 °C. The next day, a trypsin inhibitor solution was added and the tissue was warmed to 37 °C. Following this, a collagenase solution was applied to the preparation and allowed to incubate at 37 °C with light shaking for approximately 30 minutes. Gentle dissociation using a serological pipet, followed by mild centrifugation in enzyme-free culture media was used to further separate myocytes and remove the majority of red blood cells. This step was repeated twice to ensure complete removal of the collagenase enzyme. The preparation was pre-plated in a tissue culture incubator for two hours at 37 °C / 6% CO_2_ to reduce the fibroblast population. Following this pre-plating period, the myocyte rich suspension was collected and counted on a hemocytometer to determine total yield, then plated at 100K—300K confluency in DMEM (Corning 10-013-CV) complete medium containing 10% FBS (Sigma F0926), 1% non-essential amino acids (Sigma, M7145), and 1% antibiotic solution (Corning 30-004-CI). The NRVM cultures were incubated undisturbed at 37 °C and 6% CO_2_ for 24 hours to allow cellular adhesion to the coverslips. After the initial 24 hour tissue culture period, the media was removed, cultures were washed twice in PBS, followed by replacement of culture media. Cells in wells destined to express genetically encoded mitochondrial Ca^2+^ indicator were infected with an adenoviral construct (Vector Biolabs) containing the LAR-GECO1.2 plasmid obtained from Addgene (www.addgene.org) and developed by Wu et al. [10]. Following 24 hours of infection, the cultures were double washed again with PBS and fresh culture media was added. The genetically encoded indicator was expressed for an additional 24 hours before experiments commenced. All experiments were completed between 3–4 days after plating.

### Anoxia/Reoxygenation protocol

Prepared NRVM coverslips were placed into a custom perfusion bath and allowed to equilibrate for at least 20 minutes (baseline). The perfusion solution for both baseline and “reperfusion” contained 126 mM NaCl, 4.4 mM KCl, 1 mM MgCl_2_, 11 mM glucose, 24 mM HEPES and was titrated with NaOH to pH = 7.4. In the simulated “ischemic” solution, [K^+^] was increased from 4.4 to 8.8 mM, pH was decreased from 7.4 to 6.5, and glucose was replaced with 2-deoxyglucose (11 mM). All solutions contained 0.5 mM Probenecid to aid in the retention of AM dyes. The “ischemic” cocktail was gassed with nitrogen for at least 1 hour to deplete oxygen. Several minutes before the anoxic period, sodium-hydrosulfate (1 mM) was added to eliminate any remaining oxygen. Upon completion of the baseline period, “ischemia” commenced. During ischemic episodes we monitored fluorescence images and waited until we observed the loss of Ca transient, a decrease of ΔΨ_m_-sensitive fluorescence to below 50% of the average baseline level, and myocyte shrinking and blebbing. These events occurred in different NRVM monolayers between 15 and 30 min of “ischemia”. Once the observable signs of anoxic effects were complete, coverslips underwent “reperfusion” for at least 1 hour. Throughout the entire protocol, temperature was maintained at 37 ± 1°C. Cells were paced at 0.5–1 Hz beginning at baseline, and only coverslips which retained Ca^2+^ transients for the full baseline period were accepted to move forward with the simulated I/R protocol.

### Intervention groups

#### Sarcoplasmic reticulum

Experiments in the *Control* group were performed with the simulated I/R protocol as described without any additional drugs or interventions. To examine the contribution of the sarcoplasmic reticulum (SR) as the possible source of abnormal increase in cytoplasmic ([Ca^2+^]_Cy_) and mitochondrial ([Ca^2+^]_Mi_) Ca^2+^ concentrations, SR Ca^2+^ stores were depleted by a combination of ryanodine (5 μM) and thapsigargin (1 μM) applied 15 min before the onset of simulated I/R and maintained throughout the whole experiment (*Rya-Thap* group).

#### Sarcolemma permeation

The mechanism of massive Ca^2+^ influx across the sarcolemma during simulated I/R was investigated in three experimental series. The first included the application of 3-min pulses of baseline solution with nominally zero [Ca^2+^] before I/R, and after a perceptible onset of [Ca^2+^]_Cy_ overload during reperfusion (*Zero-Ca* group). In the second series we applied Ni^2+^ (5 mM) to block Na^+^-Ca^2+^ exchange (NCX) [11] (*Nickel* group). In the third series ZnCl_2_ (10 μM) was added to the perfusate for at least 5 mins before the onset of simulated I/R, and in some experiments was kept in the solution for the remainder of the experiment, while in others was resumed directly upon reperfusion (*Zinc* group). In this series NRVMs were loaded with the Zn^2+^ indicator Newport Green [12]. Baseline permeability to Zn^2+^ is very low [12] (as was confirmed by weak responses to Zn^2+^ pulses before I/R). Zn^2+^ was employed to test whether Zn^2+^ uptake coincides with Ca^2+^ uptake during reperfusion, which would suggest an opening of a non-selective pore. Also, since Zn^2+^ has a smaller hydrated radius (4.3 Å) than even the smallest SP indicators (estimated Stokes radius of at least 5.0 Å for YO-PRO1), the cellular uptake of Zn^2+^ may reveal the development of sarcolemmal pores earlier than uptake of even the smallest fluorescent probes.

#### Mitochondria permeation

To assess whether inhibiting the MPT pore would alter the timing or sequence of critical events observed under Control conditions, the MPT inhibitor, Cyclosporine A (0.2–0.4 μM), was added to all perfusion solutions beginning at 10 mins prior to the onset of I/R and remaining until the completion of the experiment (*CsA* group).

### Fluorescent indicators

To track [Ca^2+^]_Cy_ in the *Control*, *Rya-Thap*, *Zero-Ca* and *CsA* groups, NRVMs were incubated with Fluo-4 AM (9.1 μM, Thermo Fisher Scientific) in Optimem for 25 mins at room temperature previous to placement in the imaging perfusion chamber. In 3 special experiments in which cells were stained simultaneously with Fluo-4 AM and the cell-impermeable nucleic acid dye TO-PRO3, the loss of Fluo-4 was coincident with the uptake of TO-PRO3 (see S1 Fig), hence the loss of Fluo-4 could be used as the indicator of “canonical” SP during I/R. All NRVMs in these groups expressed the genetically encoded indicator, LAR-GECO1.2, to track [Ca^2+^]_Mi_. NRVMs were also stained with a ΔΨ_m_ indicator MitoView633 (10 nM, Biotium) added to the perfusate for 8 min prior to baseline imaging, and then again upon “reperfusion” for additional 6 min to replenish dye lost due to ΔΨ_m_ depolarization during the simulated ischemic period. This combination of indicators allowed for the simultaneous monitoring of [Ca^2+^]_Cy,_ [Ca^2+^]_Mi_, ΔΨ_m_, and the onset of SP (as the cellular loss of Fluo-4).

In the *Zinc* group, the cytoplasmic concentration of Zn^2+^ ([Zn^2+^]_Cy_) was monitored with a zinc-specific dye, Newport Green DCF (7.5 μM, Thermo Fisher Scientific). Rhod-2 AM (2.5 μM, Cayman Chemical) was employed to detect [Ca^2+^]_Cy_ and SP instead of Fluo-4 AM, to avoid overlap with the emission spectrum of Newport Green DCF. Both dyes were loaded by incubation in Optimem for 35 mins at room temperature, and MitoView633 was used identically to the other groups. The *Zinc* group was not infected with the LAR-GECO1.2 indicator, as it would overlap with the emission spectrum of Rhod-2. Thus, in the *Zinc* group, four parameters were also tracked, but [Ca^2+^]_Mi_ was replaced with [Zn^2+^]_Cy_. In some experiments, the SP indicator TO-PRO3 (167 nM, Life Technologies) was used as an additional method to detect SP. In spite of its overlapping spectra with MitoView633, the two indicators are spatially distinct (MitoView633 in the mitochondria, TO-PRO3 in the nucleus) and in practice TO-PRO3 uptake almost always occurred after MitoView633 loss. TO-PRO3 is normally cell impermeable and similar to the commonly used SP indicator propidium-iodide, in that it binds to nucleic acids upon entering permeable cells which amplifies the fluorescent intensity over 100-fold.

### Confocal imaging

NRVMs were placed in the perfusion bath and imaged on a Leica SP8 confocal microscope. An oil immersion 20x lens was employed for the collection of all images. Ca^2+^ transients were captured as a continuous time-series every 100 ms for 80 frames, equaling a recording total of 8 secs. The field of view (FOV) was set to a 128 x 128 pixel resolution and a 4.5x magnification with the 20x objective, along with an imaging speed of 1400 to achieve the stated continuous time resolution. For tracking all parameters throughout the full experimental period, time-series were utilized again with a frame rate of either 30 secs or 1 min depending on the experiment. These frame rates were chosen as the best balance between better time resolution and avoiding phototoxicity and bleaching due to excessive laser exposure induced by higher frame rates, such as every 15 sec. The spatial resolution of 512 x 512 pixels at a magnification of 1.3x yielded a 450 x 450 μm FOV. During a time-series, if there appeared to be a slight drift in imaging plane then the series was stopped and the plane was quickly refocused to minimize the time between the acquired series.

All long time-series images were obtained in three channels sequentially. In the first channel, either Fluo-4 or Newport Green was excited with a 488 nm wavelength laser and the emitted signal was filtered through the Leica SP8 prism. Light between 491 and 551 nm was collected with a HyD detector. The second channel, also collected with a HyD detector, imaged either LAR-GECO1.2 or Rhod-2 and was excited with a 561 nm laser and band passed between 566 and 630 nm. In the third channel MitoView633, and sometimes additionally TO-PRO3, were excited at 633 nm and long pass filtered to a PMT detector at 643 nm. Using the Leica SP8 prism technology and imaging each channel sequentially, as opposed to simultaneously, avoided possible crosstalk between signals and ensured quality intensity curves upon analysis.

### Data analysis methods and criteria

We randomly selected 5 to 7 NRVMs per plate which met the criteria of (1) being a myocyte (exhibiting rhythmic Ca^2+^ transients at baseline); (2) having clearly demarcated cell boundaries to allow for accurate cell segmentation; (3) having overt signs of “ischemic” effects including at least 50% loss in the ΔΨ_m_ and cell shrinking, and (3) surviving within 6 minutes of “reperfusion”, as demonstrated by at least 50% recovery in the ΔΨ_m_ during MitoView633 restaining combined with the lack of SP (i.e., retainment of Fluo-4 inside cells). These criteria were imposed due to the fact that these characteristics are observed in the whole heart model [7], and the aim here was to replicate whole heart events so the findings could be applied in entirety and not as a model specific phenomenon. Once acceptable myocytes were identified, they were manually segmented across all images of a time-series, and the area and mean fluorescence for each selected cell in each frame was calculated using ImageJ software.

The area and mean fluorescence were then multiplied together to determine the total fluorescence per cell. In *Control*, *Rya-Thap* and *CsA* groups the signals were normalized to the range between minimum and maximum values that occurred during the “reperfusion” phase. Specifically, the fluorescence values during reperfusion were computed as (F-F_Min_)/(F_max_-F_min_), where F is the actual level of fluorescence reported by the microscope; F_Min_ is the lowest fluorescence level during reperfusion; and F_Max_ is the highest fluorescence level during reperfusion. We defined the time of a critical increase in [Ca^2+^]_Cy_ (T_CaCy_) and a critical increase in [Ca^2+^]_Mi_ (T_CaMi_) as the time when the respective signals (Fluo-4 or LAR-GECO1.2 fluorescence) increased above the 50% level between the minimum and maximum. We defined the time of MPT (T_MPT_) as the decrease of MitoView633 fluorescence below the 50% level. Finally, we defined the time of SP (T_SP_) as the time when Fluo-4 fluorescence decreased below the 50% level. We interpreted the loss of the Fluo-4 signal during “reperfusion” as evidence of dye leakage from cells through sarcolemmal pores. This interpretation was confirmed in separate experiments, in which the loss of Fluo-4 signal during “reperfusion” correlated well with the uptake of the normally cell-impermeable dye, TO-PRO3 (see S1 Fig).

For the *Zinc* group, we also defined T_CaCy_ and T_SP_, but additionally we defined T_ZnCy_ as the time of abnormal uptake of Zn^2+^. However, here we used a slightly different approach. First, we used Rhod-2 to detect abnormal uptake of Ca^2+^ into cells instead of Fluo-4, for spectral compatibility with the other two fluorophores, Newport Green and TO-PRO3. Unlike Fluo-4, a significant fraction of Rhod-2 may redistribute into mitochondria [13] although the cytoplasmic fraction is always large [14]. However, from Control experiments we determined that the mitochondrial Ca^2+^ uptake, as indicated by an increase in LAR-GECO1.2 fluorescence, always lags behind the cytoplasmic Ca^2+^ uptake as indicated by Fluo-4 (see Results). Hence, the earliest detectable increase in Rhod-2 fluorescence should reflect mostly [Ca^2+^]_Cy_, but even if it reflects some weighed combination of [Ca^2+^]_Cy_ and [Ca^2+^]_Mi_, this still would indicate a net Ca^2+^ influx into the cell, which was the main event of interest. Also, in the *Zinc* group we first searched for the minimum of Rhod-2 signal during “reperfusion”, and normalized the signal to the range between that minimum and the maximum value during “reperfusion”. Lastly, for all the events (T_CaCy_, T_ZnCy_ and T_SP_), we set the threshold of detection at 10% level of the normalized signal, as opposed to 50% level used in other series. The low threshold was chosen to facilitate early detection of Zn^2+^ and TO-PRO3 uptake, which developed in time rather slowly, and by visual inspection was deemed to be robust enough not to produce false-positive detections.

### Statistics

All values within groups are presented as the mean ± standard error of means. Quantification plots portray a variety of data including 1) the detected values according to the analysis criteria, 2) box and whiskers plots which portray median values, the upper and lower quartiles, and the highest and lowest observations excluding outliers, and 3) mean diamond plots which incorporate the mean values and 95% confidence intervals. These plots were generated with JMP trial software (www.jmp.com). Statistical tests included Student t-tests and one-way ANOVA with Newman-Keuls post-hoc test for pairwise comparisons (XLSTAT by Addinsoft, www.xlstat.com), as indicated in specific figure legends. Differences with p < 0.05 were considered statistically significant.

## Results

### Validation of LAR-GECO1.2 localization and the viability of infected NRVMs

**Fig 1:**
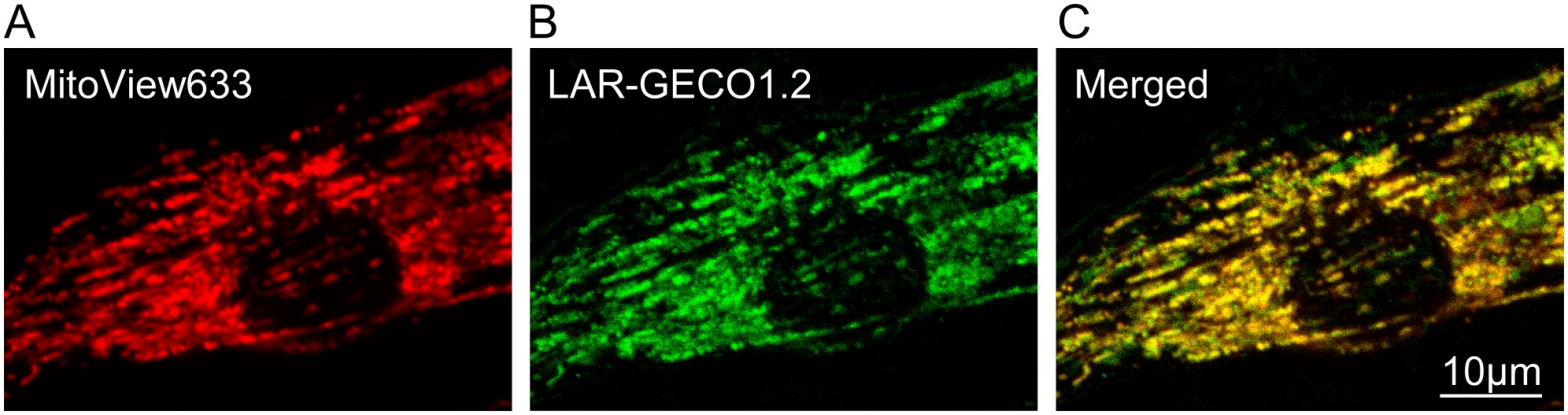
Evidence for localization of LAR-GECO1.2 to mitochondria in transfected NRVMs. **A-C,** high resolution images of the ΔΨ_m_ dye MitoView633 (**A**), the genetically encoded mitochondrial [Ca^2+^] indicator LAR-GECO1.2 (**B**), and their merged image (**C**). The images support predominantly mitochondrial localization of LAR-GECO1.2. Note the abundance of yellow (and lack of red) areas in **C**, suggesting that LAR-GECO1.2 is expressed homogeneously within mitochondria in infected cells. Green areas here indicate mitochondria that are expressing LAR-GECO1.2, but undergo ΔΨ_m_, flickering, possibly resulting from laser exposure due to imaging. This further implies that in spite of ΔΨ_m_ depolarization, LAR-GECO1.2 is still retained in the mitochondria.

Before beginning experiments with the simulated I/R, the infected NRVM model was tested to ensure the [Ca^2+^]_Mi_ indicator, LAR-GECO1.2, was properly expressed and localized to the mitochondria. To ensure appropriate localization, NRVMs expressing LAR-GECO1.2 were stained with the ΔΨ_m_ dye, MitoView633, and co-localization of the two indicators was determined using high resolution imaging. Fig 1 confirms that LAR-GECO1.2 is indeed expressed homogeneously throughout all mitochondria within the imaged cell, and exhibits a signal strong enough for experimental purposes.

LAR-GECO1.2 appears to be highly co-localized with MitoView633, as seen in the yellow areas of Panel C, and thus is expressed exclusively in mitochondria without other offhand targets within the cell. The limited green areas in Panel C that are devoid of MitoView633 staining represent temporarily depolarized mitochondria, and reflect mitochondrial depolarization-repolarization cycles (flicker) sometimes observed at baseline. In sham experiments, the cell-averaged level of LAR-GECO1.2 fluorescence remained relatively constant over a full 2-hour test period (S2 Fig). A known problem of GECO family of indicators, which includes LAR-GECO1.2, is a significant pH sensitivity, such that the fluorescence decreases at lower pH [15] (see also S3 Fig). Our use of LAR-GECO1.2 to monitor [Ca^2+^]_Mi_ during “reperfusion” was based on the assumption that after recovery of pH in the reperfusion solution to 7.4, any further significant increase in LAR-GECO1.2 fluorescence should be mostly due to an increase [Ca^2+^]_Mi_ rather than an increase in pH. Increase in intra-mitochondrial pH (alkalization) is expected when mitochondria hyperpolarize and increase the pH gradient across the inner mitochondrial membrane. However, during “reperfusion” we observed a large increase in LAR-GECO1.2 signal just before the loss of ΔΨ_m_, and this increase was sustained after the loss of ΔΨ_m_ when the mitochondrial matrix is expected to become more acidic. Hence, it seems that under the tested conditions, changes in LAR-GECO1.2 fluorescence reflected a true [Ca^2+^]_Mi_ increase, even if attenuated due to expected acidification of depolarized mitochondria.

**Fig 2:**
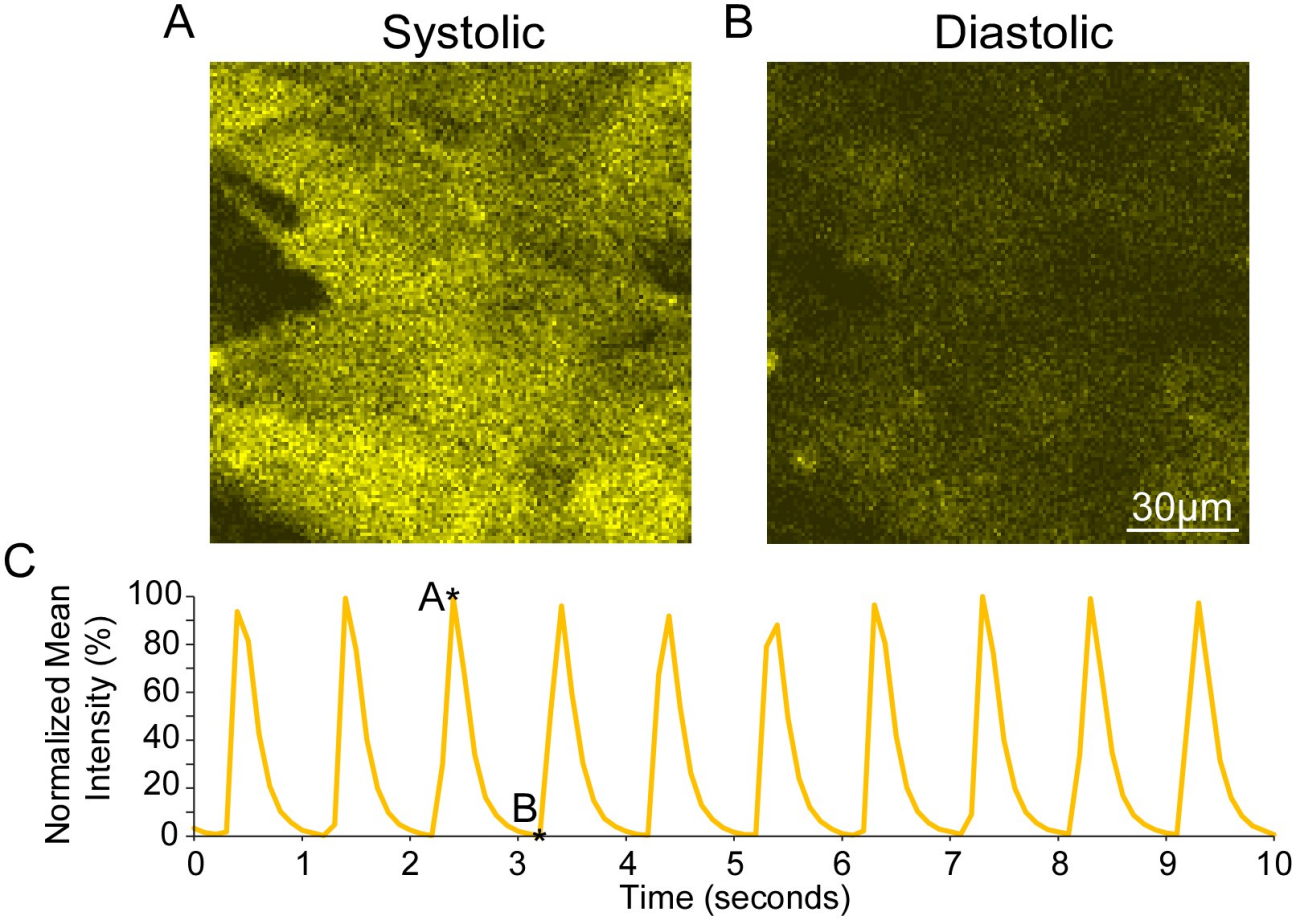
Paced [Ca^2+^] transients in infected NRVMs. Images from infected NRVMS loaded with the [Ca^2+^]_Cy_ indicator, Fluo-4 AM, during the peak (**A**) and the trough (**B**) of the [Ca^2+^] transient. Monolayers were paced at 1 Hz and imaged continuously every 100 msec. The resulting [Ca^2+^] transients (Panel C) show 1:1 capture, verifying that infected cells are viable, appear to have normal [Ca^2+^] cycling, and are able to sustain pacing.

The viability of infected NRVMs was also examined to guarantee that they functioned similarly to non-infected NRVMs and preserved normal Ca^2+^ handling. Coverslips were incubated with Fluo-4 AM to detect [Ca^2+^]_Cy_, and subsequently placed in the imaging perfusion chamber. Cells were paced at 1 Hz and continuously imaged every 100 ms. The resulting Ca^2+^ transients can be seen in Fig 2C, with 1:1 pacing capture and robust transient upstrokes. The Fluo-4 signal during the peak and trough of the Ca^2+^ transient is shown in Fig 2A and 2B respectively. In sham experiments, the infected NRVMs maintained Ca^2+^ transients for the entirety of a 2-hour experimental duration, and had not undergone any detectable SP (see S2 Fig). Due to these positive functional and viability results, NRVMs expressing LAR-GECO1.2 were deemed adequate for use in the simulated I/R experiments.

### Consistent chronology of critical events in NRVMs during “reperfusion”

In *Control* experiments, the NRVMs transfected with LAR-GECO1.2 were also loaded with Fluo-4 and MitoView633 and were subjected to 15–30 min of simulated ischemia, followed by 60 min of “reperfusion”. During simulated ischemia, NRVMs exhibited various degrees of ΔΨ_m_ dissipation and cellular shrinkage, but the vast majority of cells survived until reperfusion. However, the vast majority of cells surviving “ischemia” underwent critical transitions including full ΔΨ_m_ loss and SP later in “reperfusion”. Importantly, the dynamics of the critical events varied between myocytes which underscored the necessity to track these events on a cell-by-cell basis.

**Fig 3:**
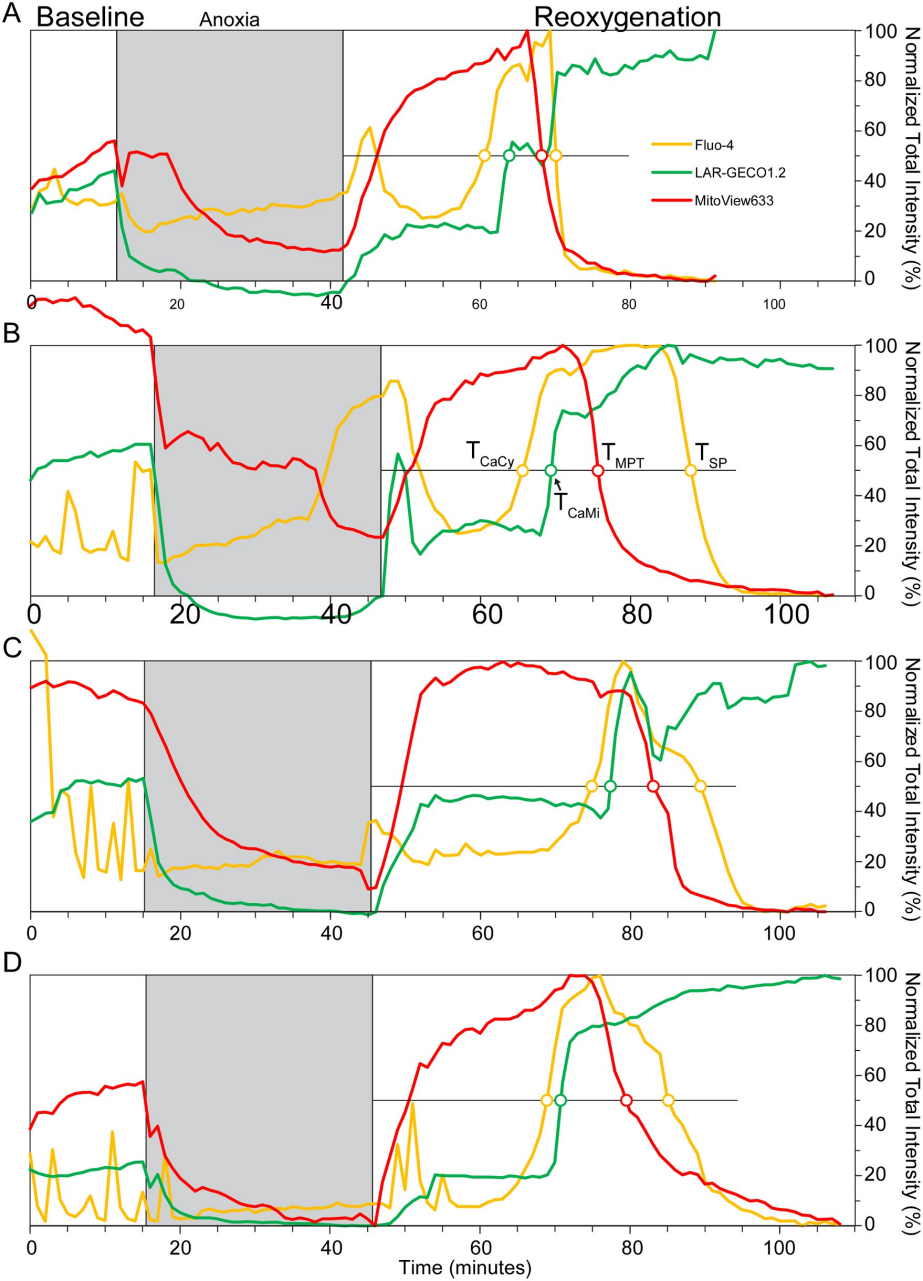
Chronology of post-reperfusion events in *Control* cells. Data from 4 separate representative *Control* cells (**A**-**D**) imaged throughout the full experimental duration at a rate of 1 frame per min. Yellow, green, and red curves show total per-cell fluorescence of Fluo-4 ([Ca^2+^]_Cy_), LAR-GECO1.2 ([Ca^2+^]_Mi_) and MitoView633 (ΔΨ_m_), respectively. All curves are normalized to their respective minimum and maximum intensity values during the “reperfusion” period. The anoxic phase is depicted by the grey box, while the thin horizontal black line during “reperfusion” indicates the 50% level used to determine the associated time points of events (colored open circles, as labeled in Panel **B**). The time points of T_CaCy_ and T_CaMi_ were estimated at the 50% rise in the fluorescence of Fluo-4 and LAR-GECO1.2, respectively, whereas T_MPT_ and T_SP_ were estimated as the 50% decrease of MitoView633 and Fluo-4 fluorescence, respectively (the latter indicating the dye efflux from the cell). Notice that in all 4 cells, this sequence of events remains the same regardless of when in reoxygenation they begin to occur.

**Fig 4:**
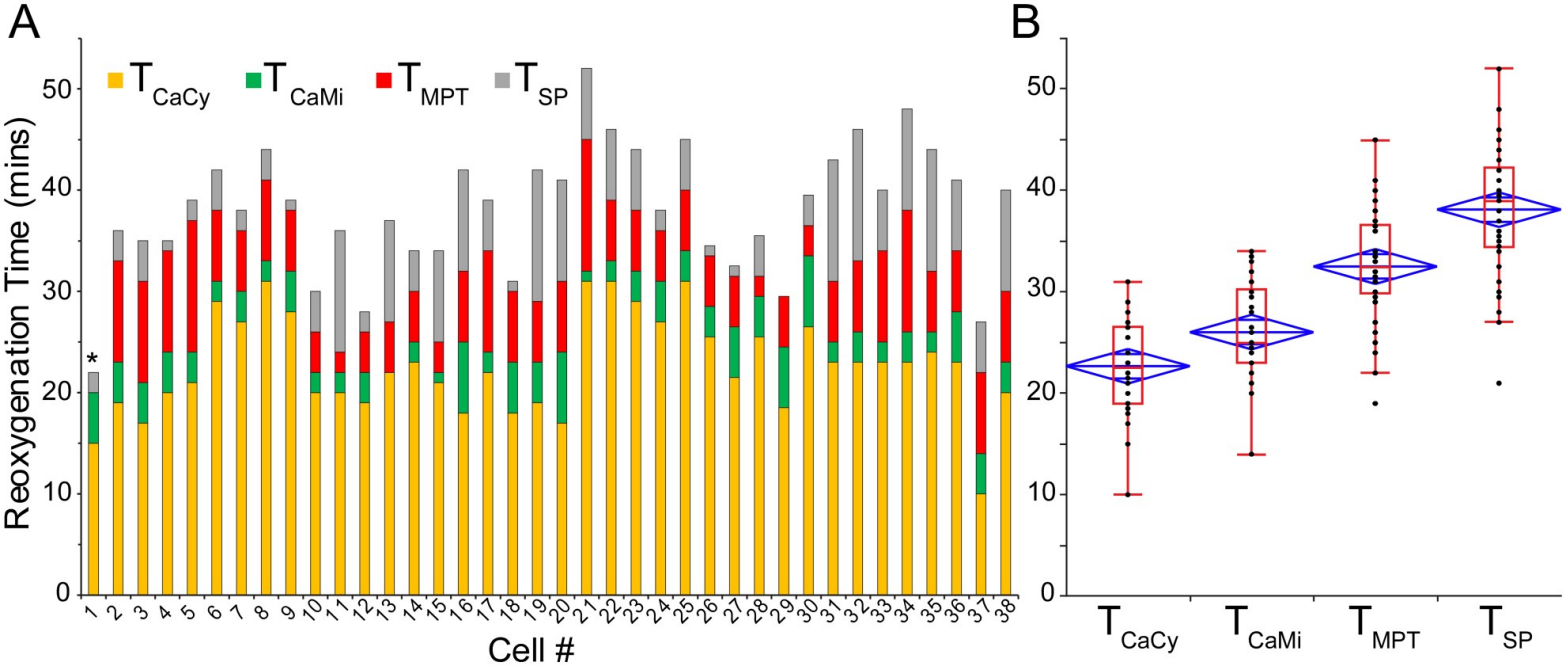
Quantification of critical events timing in the *Control* group. **A**, the timing of detected events (T_CaCy_, T_CaMi_, T_MPT_, T_SP_) with respect to the onset of “reperfusion” for all analyzed Control cells. The style is such that each color indicates the time interval between the specific event (as indicated) and the previous event. For T_CaCy_ the previous event is the onset of “reperfusion”. In all cells [Ca^2+^]_Cy_ increase was the first observed event, followed by [Ca^2+^]_Mi_ increase, then MPT, and finally SP (as detected by loss of Fluo-4). This sequence was preserved in every cell in the Control group, with the exception of cell #29, where MPT and SP occurred at the same time (hence the absence of a grey bar), and also cell #1 (indicated by an asterisk) where both MPT and SP occurred very quickly and, according to the detection criteria, SP occurred 1 min ahead of MPT. **B**, the spread of detected time points for each event (black dots) overlaid with the associated box and whiskers plot (red) and mean diamond plot (blue). The average time of T_CaMi_, T_MPT_, and T_SP_ each were statistically different from the respective previous event (all p < 0.05).

Representative curves from four different *Control* NRVMs are shown in Fig 3. (The decrease in LAR-GECO1.2 signal during simulated ischemia is attributed to imposed acidic conditions and is ignored). Note the considerable variability in [Ca^2+^]_Cy_ dynamics during simulated ischemia and during the first 5–7 min of “reperfusion”, which was present even among NRVMs from the same monolayer. An initial [Ca^2+^]_Cy_ rise could occur during ischemia (Fig 3B), or immediately upon reperfusion (Fig 3A, 3C and 3D). It is of interest, however, that regardless of the presence or the absence of these early [Ca^2+^]_Cy_ events, they were followed by a period of an apparent tranquility until the secondary [Ca^2+^]_Cy_ increase invariably occurred, heralding the onset of cellular transition to death. It can be seen that despite variability in the dynamics of specific signals among the presented cells, there was a consistent sequence of critical events, in the order of T_CaCy_ < T_CaMi_ < T_MPT_ < T_SP_. This sequence was conserved in all but one analyzed *Control* cell (see Fig 4; asterisk denotes the exception). [Ca^2+^]_Cy_ overload occurred at an average of 22.7 ± 4.9 mins of “reperfusion”, followed by [Ca^2+^]_Mi_ overload approximately 3.4 ± 1.7 mins later, then MPT at 32.5 ± 5.5 mins, and finally SP at 38.1 ± 6.3 mins. T_CaMi,_ T_MPT,_ and T_SP_ were found to be significantly separated from their respective previous event (all p values < 0.05 by paired t-test). The application of CsA (0.2–0.4 μM) (CsA group), an MPT inhibitor which decreases the MPT pore sensitivity to Ca^2+^, did not change the timing or order of the critical events (S4 Fig).

Summarizing, in this model we detected a [Ca^2+^]_Cy_ overload event which is separate from the [Ca^2+^]_Cy_ fluctuations occurring immediately upon reperfusion, and which invariably leads to [Ca^2+^]_Mi_ overload, MPT and SP. We further tried to sort out whether the observed increases in [Ca^2+^]_Cy_ and [Ca^2+^]_Mi_ are due to Ca^2+^ release from SR, or due to a Ca^2+^ flux across the cell membrane.

### Sarcoplasmic reticulum Ca^2+^ depletion accelerates the timing of critical events in reperfusion

**Fig 5:**
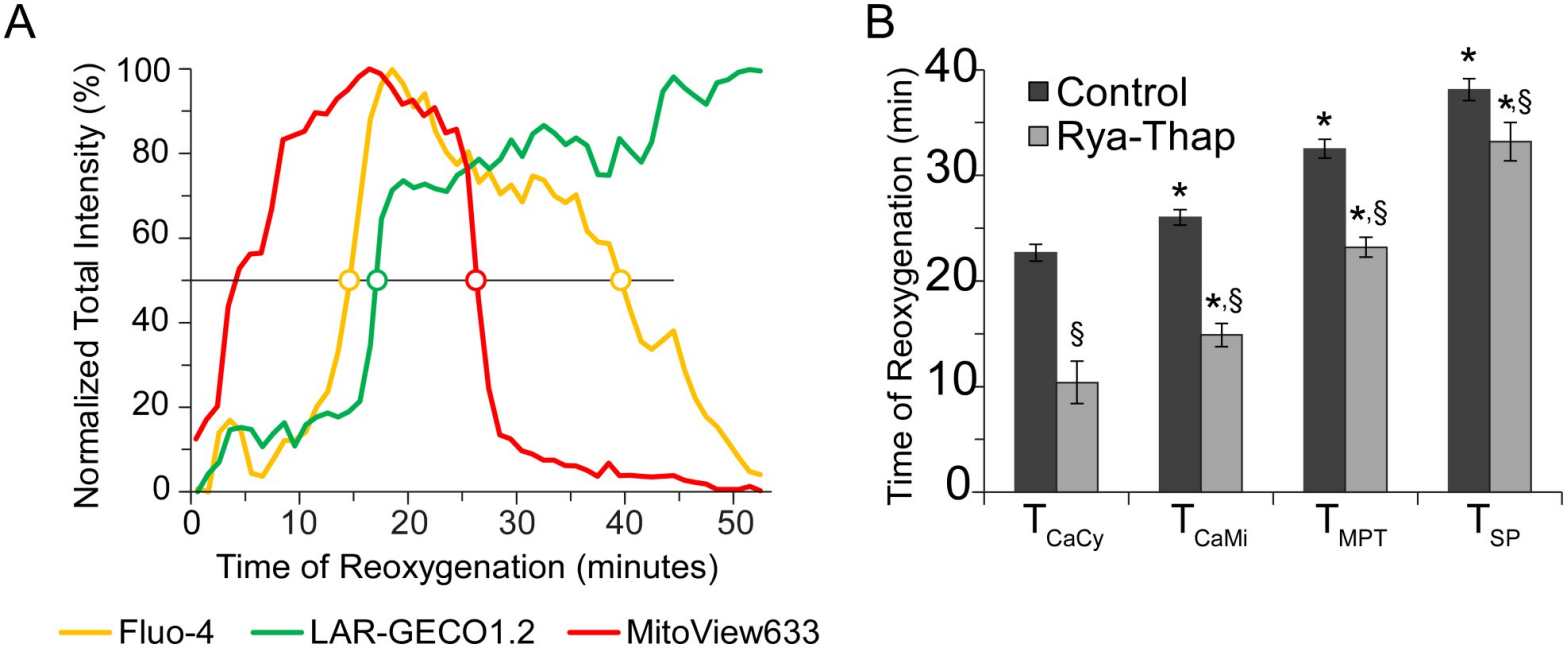
Effect of sarcoplasmic reticulum depletion by ryanodine/thapsigargin. **A**, fluorescence intensity curves during “reperfusion” from a representative cell in the Rya-Thap group. All labeling and notations are the same as in Fig 3, but only “reperfusion” phase is shown. **B**, when quantified, the sequence of critical events in the Rya-Thap group (grey bars) is the same as in Control group (black bars), however, each event in the Rya-Thap group occurred at a significantly earlier time point of “reperfusion” than in Control group. *, p < 0.05 as compared to the timing of previous event in the same group (paired t-test); §, p < 0.05 as compared to the timing of the same event in different group (unpaired t-test).

Previously, a Ca^2+^ release from SR during reperfusion/reoxygenation was implicated in mitochondrial Ca^2+^ loading, MPT, and cell death [4]. To test the possible role of a large Ca^2+^ release from the SR as the trigger of the observed sequence of critical events, the SR was depleted with continuous perfusion of ryanodine (5 μM) and thapsigargin (1 μM), keeping ryanodine receptor channels in a continually open state while also inhibiting Ca^2+^ uptake into the SR by blocking SERCA2a (*Rya-Thap* group). Panel A in Fig 5 shows normalized intensity curves from a representative *Rya-Thap* cell during reperfusion, analyzed identically to cells in the *Control* group. Most notably, the major sequence of events remained the same as observed in *Control*, with [Ca^2+^]_Cy_ overload occurring first, followed by [Ca^2+^]_Mi_ overload, then MPT and SP. When quantified and compared to Control (Panel B), the sequence of critical events indeed remained the same as seen in *Control*, with T_CaMi,_ T_MPT,_ and T_SP_ occurring significantly later than the respective previous event (indicated by an asterisk, p<0.05). Interestingly, in spite of the retention of the event sequence, *all* critical events in the *Rya-Thap* group occurred significantly earlier than their *Control* counterpart (indicated by a §, p<0.05). The cause and/or meaning of this observation will be deliberated in the Discussion section.

### Cytoplasmic and mitochondrial Ca^2+^ overload during reperfusion is due to Ca influx across the sarcolemma

**Fig 6:**
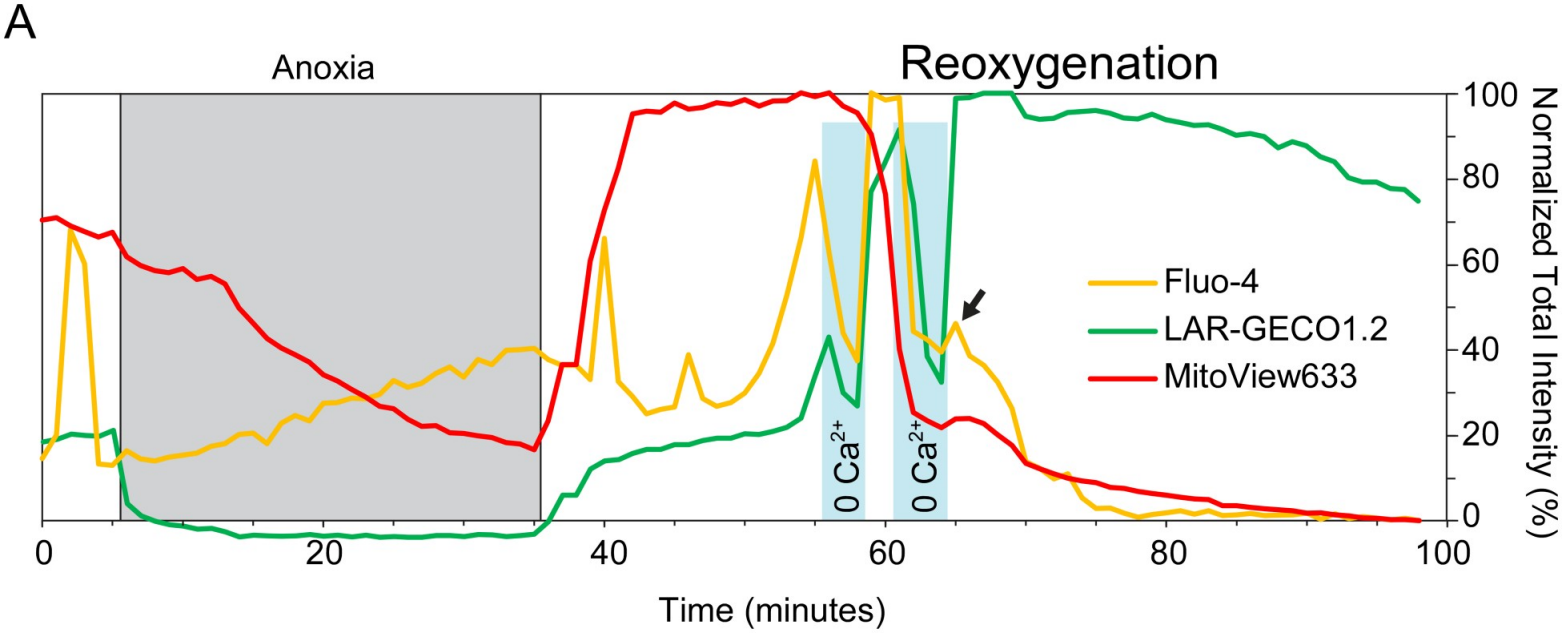
[Ca^2+^]_Cy_ and [Ca^2+^]_Mi_ directly follow changes in extracellular [Ca^2+^] after the start of [Ca^2+^]_Cy_ overload. A representative example of responses to pulses of nominally 0 [Ca^2+^] applied after the start of noticeable Ca^2+^ overload during “reperfusion” (Zero-Ca group). Blue shades indicate the time of perfusion with nominally 0 [Ca^2+^]. Other notations are the same as in Fig 3. Note that both [Ca^2+^]_Cy_ and [Ca^2+^]_Mi_ strictly follow the changes in extracellular [Ca^2+^], with apparently symmetric fluxes in both inward and outward directions. The fact that after the second pulse of 0 Ca^2+^ the [Ca^2+^]_Cy_ signal does not return to overloaded levels (arrow), indicates that at this point the sarcolemma becomes permeable to [Ca^2+^]_Cy_ indicator, Fluo-4, which leaks out of the cell preventing further ability to track [Ca^2+^]_Cy_.

Since the depletion of SR did not affect the dynamics of [Ca^2+^]_Cy_ and [Ca^2+^]_Mi_ increase during reperfusion, we hypothesized that the source of these increases is an influx of Ca^2+^ ions across the sarcolemma. To prove this point, we applied 2–3 min pulses of nominally zero Ca^2+^ solution (0 Ca^2+^) after the initial phase of the [Ca^2+^]_Cy_ overload during “reperfusion” became evident during real-time monitoring in pre-selected regions of interest. Fig 6 shows representative data from a single NRVM with two 0 Ca^2+^ pulses applied after the steep rise in [Ca^2+^]_Cy_. As is apparent, both [Ca^2+^]_Cy_ and [Ca^2+^]_Mi_ drop immediately upon the application of 0 Ca^2+^, and return directly back upon the reapplication of normal [Ca^2+^]. Note the almost symmetric response of [Ca^2+^]_Cy_ to decreases and increases of [Ca^2+^]_o_, suggesting equal permeability for Ca^2+^ in both transmembrane directions.

**Fig 7:**
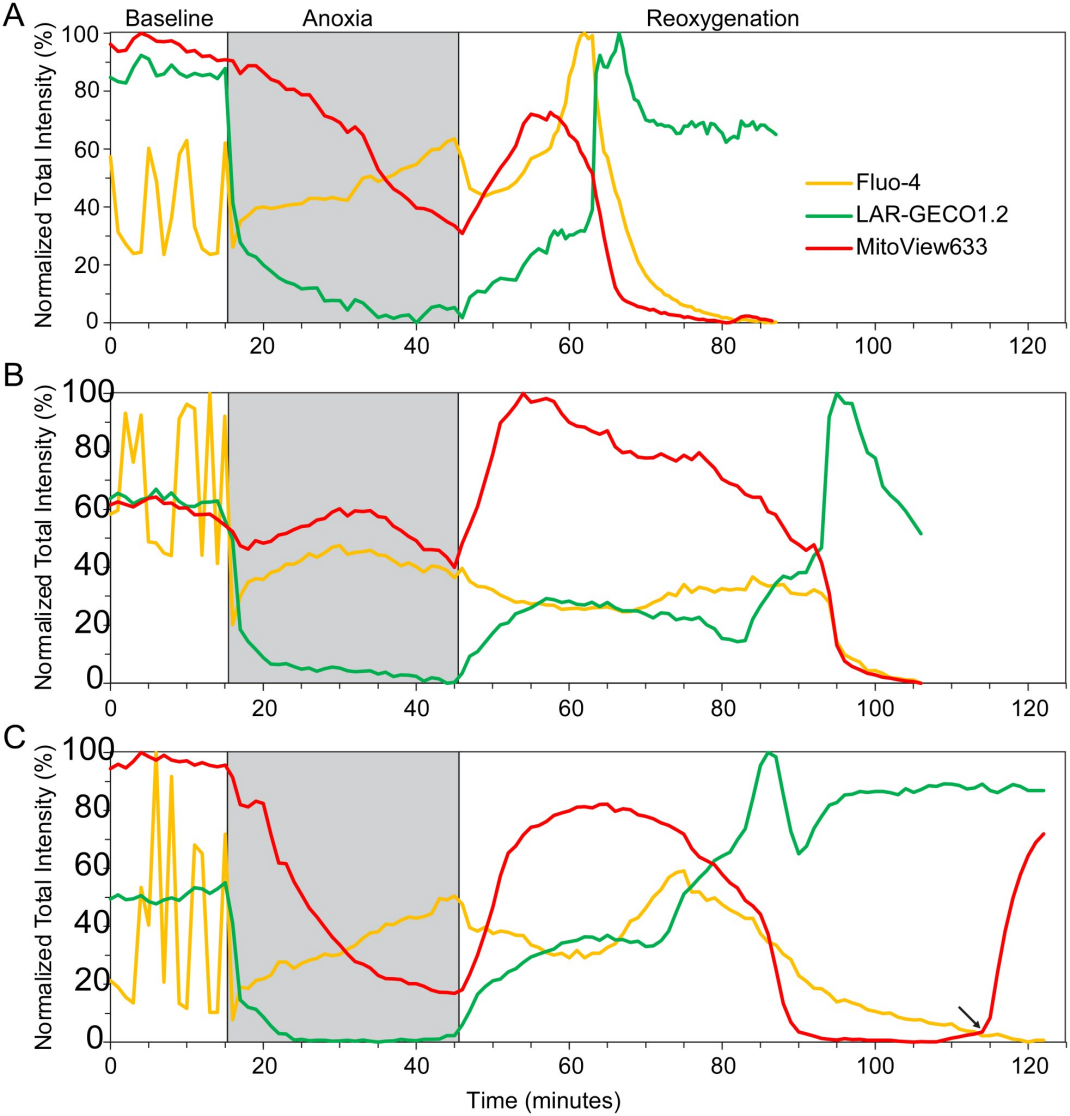
Representative examples of reperfusion events in cells subjected to 5 mM Ni^2+^. All labeling and notations are the same as in Figure, unless indicated otherwise. Note overall blunted dynamics of Fluor-4 signal during reperfusion, including the case when no apparent [Ca^2+^]_Cy_ overload ever occurred (**B**). Arrow in **C** indicate the time when TO-PRO3 was added to perfusate, immediately revealing that the sarcolemmal permeabilization had already occurred.

The symmetrical inward and outward flux of Ca^2+^ observed during “reperfusion” could be mediated Na^+^-Ca^2+^ exchanger (NCX) [16]. To investigate possible role of NCX in the [Ca^2+^]_Cy_ overload we applied 5 mM Ni^2+^ to the perfusate in the *Nickel* group. At this concentration, Ni^2+^ blocks NCX by 80% [11]. Ni^2+^ affected the events during reperfusion in multiple ways and caused astounding variability in the outcomes. Most importantly, it blunted [Ca^2+^]_Cy_ dynamics in such a way that in the majority of cells the criterion of T_CaCy_ as the time when [Ca^2+^]_Cy_ curve crossed the 50% level of the ascending phase of the dynamic range during reperfusion (see Fig 3) was not met, precluding proper comparison with *Control*. Fig 7 shows examples of diversity observed in the *Nickel* group.

Fig 7A shows a case when the events occurred similarly to the *Control* group. However, the post-reperfusion recovery of fluor-4 signal reflecting [Ca^2+^]_Cy_ was very poor, the minimum level barely scratching the 50% of the dynamic range. Also, the recovery of LAR-GECO1.2 signal, reflecting the combination of changes in [Ca^2+^]_Mi_ and pH was extremely slow as compared to *Control* cases (see Fig 3). This means that either [Ca^2+^]_Mi_, or pH, or both were affected by Ni^2+^. Fig 7B shows a case when post-reperfusion [Ca^2+^]_Cy_ overload did not occur at all. Yet, there was a gradual decrease in MitoView633 signal reflecting a slow loss of ΔΨ_m_. It was followed by fast loss of MitoView633 and Fluo-4. Almost simultaneously, there was a sharp increase in LAR-GECO1.2 signal, reflecting an abrupt increase in [Ca^2+^]_Mi_. These three events are consistent with an almost simultaneous SP and MPT, reminiscent of our observations in whole adult hearts [7]. Lastly, Fig 7C shows a case when there was a moderate increase in Fluo-4 signal during “ischemia”, followed by a poor recovery during”reperfusion” (just touching 50% level), followed by a blunted secondary increase, followed by a very slow decrease in Fluo-4 signal to the level of full disappearance. The Fluo-4 signal loss was so slow that we even doubted that it reflected true event of SP, and we applied TO-PRO3 at the moment indicated by the arrow in Fig 7C. The immediate uptake of TO-PRO3 proved that the cell was indeed permeabilized by that time. Typical for cells subjected to Ni^2+^, there was a phase of slow loss of ΔΨ_m_ followed by a phase of fast ΔΨ_m_ loss. Increase in [Ca^2+^]_Mi_ followed increase in [Ca^2+^]_Cy_ and preceded the phase of fast ΔΨ_m_ loss. Thus, qualitatively the sequence of critical events is similar to that in *Control*, but all events are somewhat blunted.

To provide a quantitative support for the notion that Ni^2+^ blunted [Ca^2+^]_Cy_ increase during reperfusion, we calculated the maximum level of Fluo-4 signal during “reperfusion” as the percent of the maximum baseline signal. (Recall that at baseline the maximal level of Fluo-4 reflected a possibly underestimated amplitude of the normal [Ca^2+^]_Cy_ transient). In analyzed *Control* cells maximal post-reperfusion level of Fluo-4 signal reached 154±6% of the maximal baseline level, whereas in analyzed *Nickel* cells it was 100±14% of the maximal baseline level (p < 0.0001 by unpaired two-tail t-test).

### Evolving sarcolemmal permeability observed in the presence of Zn^2+^

Whereas the effects of Ni^2+^ described above nominally supported the role of NCX as the conduit of post-reperfusion [Ca^2+^]_Cy_ overload, we had our doubts (explained in Discussion) and we tested an alternative hypothesis that the putative channel may be non-selective. We tested the possibility that the channel responsible for the critical increase in [Ca^2+^]_Cy_ is also permeable to ions of Zn^2+^. We chose Zn^2+^ because it is normally present in cardiac myocytes at extremely low (single nanomolar) concentrations. Also, under normal conditions, the exposure of cardiomyocytes to extracellular Zn^2+^ at 10 to 100 μM concentrations does not cause a detectable increase in [Zn^2+^]_Cy,_ and does not adversely affect cardiomyocytes apart from a slight inhibition of I_Ca,L_ [12]. In addition, Zn^2+^ is not transported by NCX [17] and does not affect Ca transport through NCX [18]. Hence, a significant increase in Zn^2+^ uptake during reperfusion would indicate abnormal cell permeability independent of NCX. Experiments were designed to determine the relative timing of permeability to Ca^2+^, Zn^2+^ and the normally cell-impermeable nucleic acid indicator, TO-PRO3, during “reperfusion” (*Zinc* group). ZnCl_2_ (10 μM) was present in the perfusate at least from the beginning of “reperfusion”. Rhod-2 AM and Newport Green DCF were used to indicate [Ca^2+^]_Cy_ and [Zn^2+^]_Cy_, respectively. TO-PRO3 fluorescence was collected in the same channel as MitoView633, since the signals could be easily separated spatially and temporally (see Methods).

**Fig 8:**
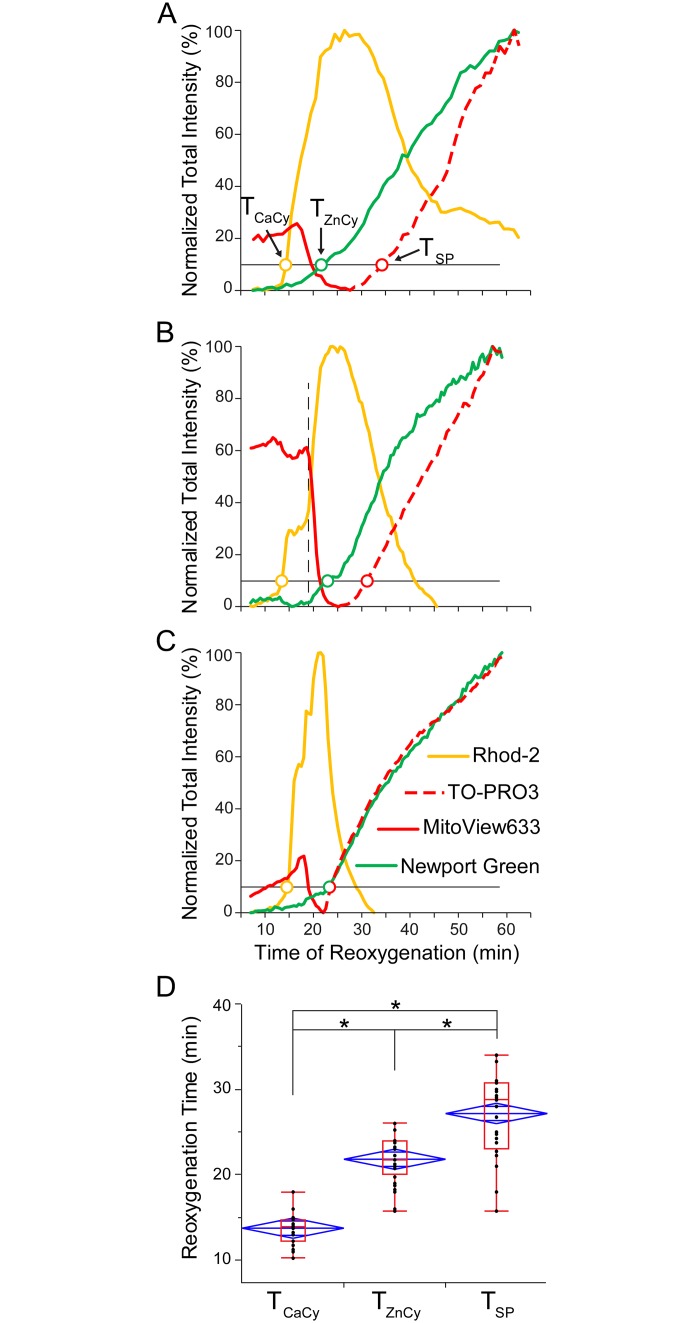
Three stages of permeability detected in the presence of Zn^2+^. **A-C**, the time course of fluorescence emitted by Rhod-2 (yellow), Newport Green (green), MitoView 633 (solid red) and TO-PRO3 (dashed red) from 3 representative cells in the *Zinc* group during “reperfusion”. The levels of fluorescence are normalized the same way as in Fig 3. Rhod-2 fluorescence mostly reflects [Ca^2+^]_Cy_, but also to some extent [Ca^2+^]_Mi_. Newport Green fluorescence reflects [Zn^2+^]_Cy_. MitoView633 and TO-PRO3 track ΔΨ_m_ and “canonical” SP, respectively. Although MitoView633 and TO-PRO3 are imaged in the same channel, the loss of ΔΨ_m_ (presumably indicating MPT) and the uptake of the normally impermeable TO-PRO3 can be distinguished because they are separated in time. The detection criteria for uptake of Ca^2+^, Zn^2+^, and TO-PRO3 was set at the 10% level of the dynamic range during “reperfusion”. Note that Ca^2+^ uptake occurs first, followed by Zn^2+^ uptake, and followed lastly by SP, with the zinc and SP events occurring at the same time in the last curve (colored open circles). In **B**, there is a ‘step’ in the [Ca^2+^]_Cy_ signal that was never observed in *Control* experiments (beginning of second phase of [Ca^2+^]_Cy_ indicated by vertical dashed line). **D**, overall, the onset of Zn^2+^ permeability occurred between the onset of permeability to Ca^2+^ and the onset of permeability to TO-PRO3. *, p < 0.0001 by one-way ANOVA with Newman-Keuls (SNK) post-hoc test for pairwise comparisons.

Fig 8A–8C portrays data from three different representative cells from the *Zinc* group. Events were detected at a 10% increase in each signal (thin black line) in the range between minimum and maximum measured during “reperfusion”. In each cell, the onset of [Ca^2+^]_Cy_ rise was detected the earliest, followed by the onset of Zn^2+^ uptake, and finally uptake of TO-PRO3 (colored open circles in Fig 8). Note that the time course of the red curve reflects two different events, where a rapid drop indicates a decrease in MitoView633 fluorescence due to ΔΨ_m_ loss (solid red line), whereas the final and sustained increase (dashed red line) indicates uptake of TO-PRO3 signifying “canonical” SP. Overall, the timing of the three permeability events varied between cells, but it was always the case that Zn^2+^ uptake started after Ca^2+^ uptake and before, or simultaneously with, TO-PRO3 uptake (T_CaCy_ < T_ZnCy_ ≤ T_SP,_ see Fig 8D). Note that this order might reflect the increasing size of the penetrating species (hydrated radius of Ca^2+^, 4.1 Å; Zn^2+^, 4.3 Å; TO-PRO3, Stokes radius ~5 Å).

**Fig 9:**
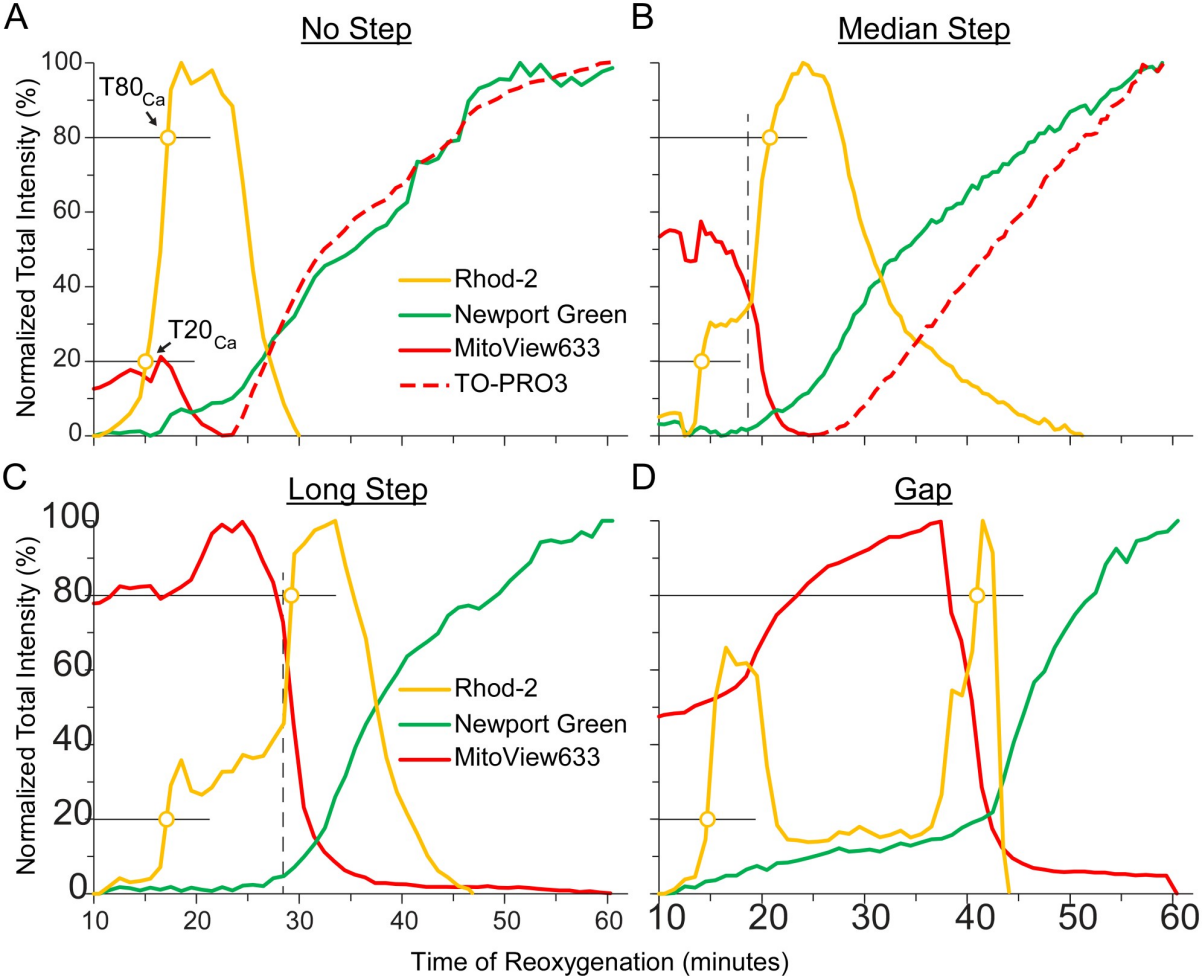
Zn^2+^-induced changes in [Ca^2+^]_Cy_ uptake dynamics. In the presence of zinc, [Ca^2+^]_Cy_ dynamics change and the analyzed cells can be divided into two groups, those with a [Ca^2+^]_Cy_ ‘step’ and those without. **A**-**D**, data from representative cells in the Zinc group portraying varying durations of the step ranging from no step (similar to Control cases), to a median and long step, and then a ‘gap’ between two phases of [Ca^2+^]_Cy_ rise. The step duration is calculated between the points where the [Ca^2+^]_Cy_ curve crosses the 20 and 80% threshold, indicated by the horizontal thin black lines and colored open circles (T20_Ca_ and T80_Ca_, respectively). This demonstrates great variability of Zn^2+^ interference in Ca^2+^ influx between different cells.

When scrutinized more closely, it became apparent that in the *Zinc* group, there exists a phenomenon in some, but not all cells, that is not present in *Control* experiments. This phenomenon is a ‘step’ or a ‘gap’ in the rising phase of the [Ca^2+^]_Cy_ curve during “reperfusion”. This ‘step’ can be detected by visual inspection and quantified as the time difference between the 20 and 80% levels of the rising phase of [Ca^2+^]_Cy_. Fig 9 shows four different cells from the *Zinc* group with varying ‘step’ durations, including a cell without a step (similar to those observed in *Control* cases), cells with a median and long step, and one with a “gap” (two phases of [Ca^2+^]_Cy_ increase separated by a period of [Ca^2+^]_Cy_ decrease). The thin black lines indicate the 20 and 80% levels of [Ca^2+^]_Cy_ rise and the colored open circles are the detected time points (denoted T20_Ca_ and T80_Ca_, respectively). The time difference between the points demonstrates how the step size may vary between different myocytes.

**Fig 10:**
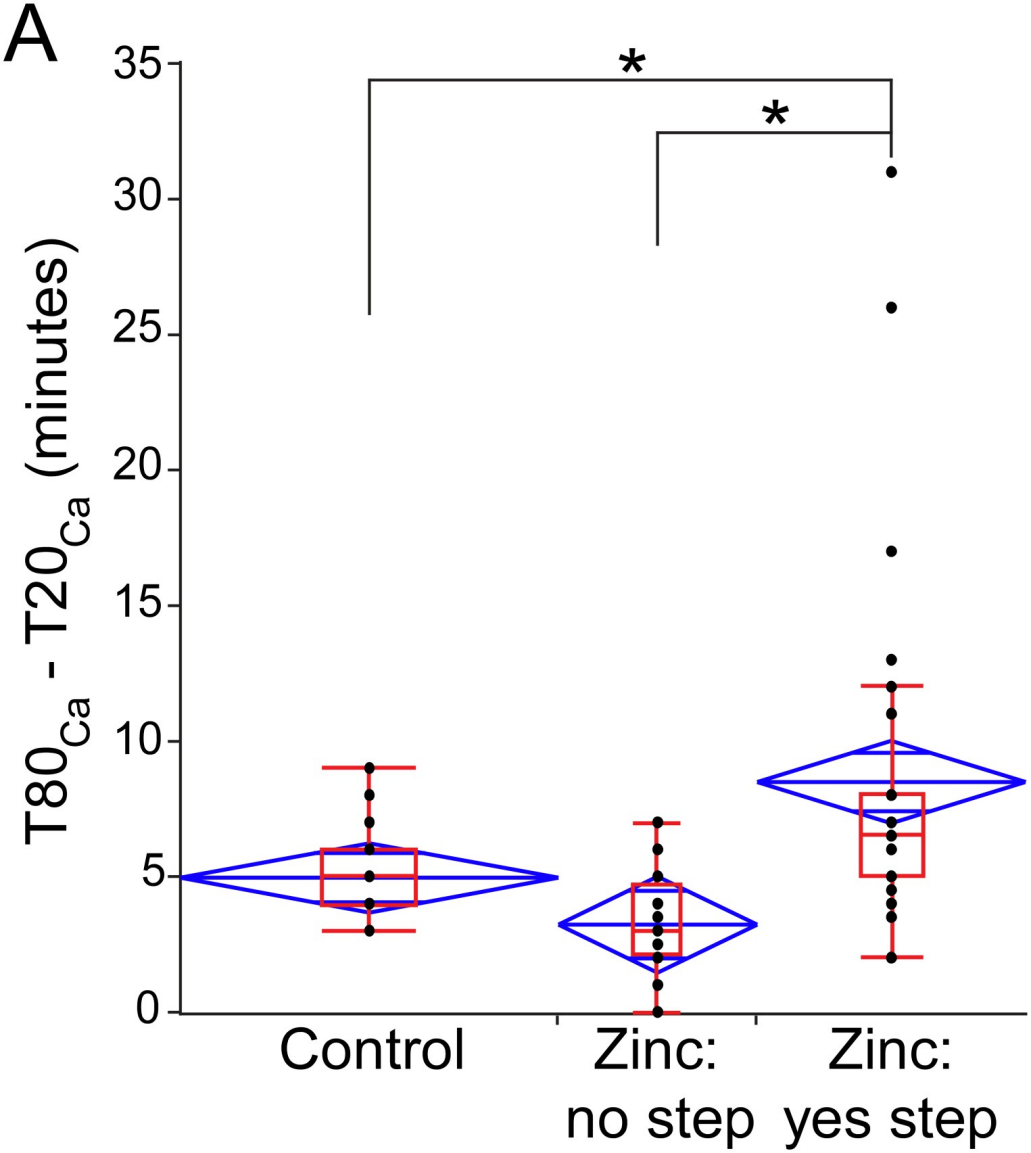
Quantification of the interval between 20 and 80% level of [Ca^2+^]_Cy_ increase during “reperfusion” (T80_Ca_—T20_Ca_) in the presence and in the absence of Zn^2+^ in the perfusate. Zinc cells without observable “steps” in [Ca^2+^]_Cy_ rise are similar to Control cells, while Zinc cells with “steps” have a significantly longer T80_Ca_—T20_Ca_ interval than either Control or Zinc without “steps” (one-way ANOVA with Newman-Keuls (SNK) post-hoc test for pairwise comparisons). Black dots indicate individual values of T80_Ca_—T20_Ca_ interval in each group, and are overlaid with both the box and whiskers plot (red) and the mean diamonds plot (blue).

Due to the great variability of Zn^2+^ effects, the time interval between T20_Ca_ and T80_Ca_ was not statistically different between the *Control* and *Zinc* groups. However, when the *Zinc* group was divided into two subgroups with and without a “step” in [Ca^2+^]_Cy_ rise, statistical analysis showed that the subset of *Zinc* cells with a “step” had a significantly longer interval between T20_Ca_ and T80_Ca_ as compared to *Control* cells and *Zinc* cells without the “step” (Fig 10).

Summarizing, experiments using Zn^2+^ in the perfusate suggest progressive sarcolemmal permeabilization, allowing for the intake of ions and molecules of increasing size as reperfusion advances. The peculiarities of mutual dynamics of Ca2^+^, Zn2^+^, and TO-PRO3 uptake suggest that all of these species permeate through the same transmembrane conduit, as discussed below.

## Discussion

The important findings of this study are threefold. First, we established a consistent chronology of critical events during reperfusion, in which cytoplasmic Ca^2+^ overload was followed by mitochondrial Ca^2+^ overload, followed by MPT, followed by the “canonical” SP. Second, we provided strong evidence that the cytoplasmic Ca^2+^ overload, the first apparently irreversible event, is due to a Ca^2+^ influx through the sarcolemma, which is uncoupled from the acute reperfusion events and is unlikely to be mediated by NCX (see below). Third, we provide evidence of specific interactions between Ca^2+^, Zn^2+^, and TO-PRO3 uptake which might suggest that their permeation during reperfusion occurs through the same common path.

### Chronology of critical events leading to cardiomyocyte death during I/R

The mechanistic understanding of I/R injury in the heart has evolved into an exceedingly complicated network of interwoven pathways which conceptually includes necrosis, necroptosis, pyroptosis, apoptosis, and autophagy (to find topical reviews, see these recent meta-reviews [19, 20]). Whether these pathways interact and converge to produce a single critical event which decides the cell fate, or there is a combinatorial explosion of death phenotypes due to variable manifestations of specific mechanisms co-occurring in the same heart, remains poorly understood. For a long time, various groups identified the opening of the MPT pore as the pivotal event in cardiomyocyte death. The MPT pore is a non-selective large channel in the outer mitochondrial membrane (with still disputed molecular composition [21]), which is triggered by a combination of increased mitochondrial [Ca^2+^], reactive oxygen species (ROS), and inorganic phosphate [22]. Opening of the MPT pore causes collapse of the mitochondrial membrane potential (ΔΨ_m_) and uncoupling of oxidative phosphorylation leading to cellular ATP depletion. In addition, it causes mitochondrial matrix swelling and rupture of the outer mitochondrial membrane resulting in the release of pro-apoptotic factors such as cytochrome c into the cytosol, thereby initiating the apoptotic pathway. Crompton and colleagues could be the first to suggest that MPT pore opening is a potential factor of acute myocardial I/R injury [22] and were the first to show that inhibition of MPT pore opening by CsA protected isolated ventricular myocytes from cell death caused by simulated I/R [23]. CsA is the most prevalent MPT pore inhibitor in experimental studies and is also the drug used to prevent reperfusion injury in clinical trials. Despite the fact that the majority (but not all [19]) experimental studies reported reduction of infarct size in CsA treated hearts/animals, the clinical trials overall show a lack of benefit when CsA was administered in patients undergoing percutaneous coronary intervention (CIRCUS trial [8]).

Different reasons were debated to explain the disappointing outcomes of the CIRCUS trial, but one important consideration here is the fact that CsA is a not a blocker, but rather a modulator, of the MPT pore. By blocking cyclophilin D, CsA reduces the sensitivity of the pore to [Ca^2+^], but cannot prevent MPT pore opening when a sufficiently high [Ca^2+^] in the vicinity of the pore is achieved [24]. In fact, MPT still occurs even in the complete absence of cyclophilin D (reviewed in [21])). Hence, pending discovery of true blockers of the MPT pore, the problem of MPT in reperfusion is reducible to the problem of a high enough level of mitochondrial Ca^2+^ overload during I/R.

Before the advent of the MPT pore theory, there was a considerable body of work supporting the notion that cytoplasmic Ca^2+^ overload during reperfusion causes cell death via excessive cardiomyocyte contraction (“hypercontracture”), leading to excessive membrane tension and mechanical rupture of the sarcolemma [25]. Clearly, sarcolemmal rupture is expected to allow a massive influx of Ca^2+^ into the cell, which could create a vicious circle further promoting hypercontracture, but also could trigger MPT. On the other hand, it was speculated long ago that the event of MPT leading to dissipation of ΔΨ_m_, can be a *cause* of hypercontracture and sarcolemmal rupture, due to a critical depletion of ATP and a release of Ca^2+^ from the mitochondrial matrix into the cytoplasm [26]. A study supporting this sequence was later published [3].

From the above one can see that the known or speculative events during reperfusion could form a double vicious circle coupled through [Ca^2+^]_Cy_. However, any vicious circle has to start from some event outside the feedback loop(s). There has to be an external trigger either for MPT, or [Ca^2+^]_Cy_ elevation, or both. To that effect, a canonical view has been developed which emphasized events occurring at the moment of restoration of oxygen and ionic imbalances at the onset of reperfusion. It is generally believed that in this setting a net Ca^2+^ influx takes place due to a coupled action of the sodium-proton exchanger and NCX in response to abrupt restoration of extracellular pH upon reperfusion (for review, see [27]). There is a theory that this excessive Ca^2+^ influx leads to SR Ca^2+^ overload and that spontaneous Ca releases from the SR facilitate mitochondrial Ca^2+^ overload through a direct Ca^2+^ cross-talk between SR and mitochondria, leading to MPT [4]. Another important acute post-reperfusion event is a burst of reactive oxygen species (ROS) occurring due to a rapid oxidation of NADH [28] or succinate [29] by a possibly damaged [30] electron transport chain. ROS elevation per se is sufficient to induce MPT. Importantly, these events are expected to occur within minutes of reperfusion [30]. Traditionally, it is also believed that MPT should occur within minutes of reperfusion, mostly based on a single line of work demonstrating that the largest increase in mitochondrial uptake of deoxyglucose, which is normally unable to cross the inner mitochondrial membrane, occurs between 2 and 5 minutes of reperfusion [31]. However, confocal imaging studies in whole hearts, using an abrupt and irreversible loss of ΔΨ_m_-sensitive fluorophores in discrete myocytes as an indicator of MPT, showed that these events spread over a period of time from tens of minutes to hours [7, 9, 32]. At least some of those events cannot be explained by Ca^2+^ overload and/or ROS occurring upon restoration of perfusion and re-introduction of oxygen.

The findings of the current study are in line with prior observations in whole hearts [7, 9, 32] that the critical cellular events are not strictly coupled to the moment of reperfusion. While there were cells which simply did not survive the period of simulated ischemia, the majority of cells underwent critical transitions 15–25 min after the restoration of normal perfusion. Although we observed transient [Ca^2+^]_Cy_ elevations consistent with an activation of NCX in the first minutes of “reperfusion” (see Fig 3), those did not directly lead to other critical events. Regardless of [Ca^2+^]_Cy_ dynamics at the end of “ischemia” and beginning of “reperfusion”, which varied among cells, it was the “secondary” increase in [Ca^2+^]_Cy_ occurring after a latent period, which led to a consistent sequence of critical events. This sequence affirms the notion that an increase in [Ca^2+^]_Cy_ is the primary event of cell damage during reperfusion, and that it is a necessary and sufficient trigger of MPT. This sequence agrees with the sequence of events found in mouse hearts subjected to anoxia/reoxygenation [9], but is different from our prior observations in whole rabbit hearts subjected to global ischemia/reperfusion [7]. In our previous study the event of MPT was virtually simultaneous with the event of SP, and administration of CsA led to SP being ahead of MPT [7]. In our current study MPT clearly preceded SP, with no effect of CsA on the sequence of events. Hence, whereas in our previous study we could implicate the occurrence of large non-selective pores in the sarcolemma in cellular Ca^2+^ overload leading to MPT, in the current study we cannot, knowing that the critical increase in [Ca^2+^]_Cy_ occurred several minutes before the “canonical” membrane permeabilization (as indicated by cellular uptake of TO-PRO3 or/or cellular loss of a Ca^2+^ indicator). We will now discuss the possible source of the [Ca^2+^]_Cy_ increase in reperfusion, and whether findings of our current study can be reconciled with earlier observations, including our own.

### The direction of pathological Ca^2+^ fluxes during reperfusion

As mentioned above, different prior studies have supported different directions of Ca^2+^ shifts between various cellular compartments during reperfusion. It is universally accepted that a net influx of Ca^2+^ into cells upon reperfusion is a critical component of I/R injury. However, the sources, the sinks, and the conduits for abnormal Ca^2+^ transport during reperfusion are not well established. The canonical view implicated reverse-mode NCX as a major source of Ca^2+^ influx into the cell during reperfusion [27]. Ca^2+^ ions entering cells should redistribute between three major compartments, the cytoplasm, mitochondria, and SR, assuming for simplicity that the Ca^2+^ absorbing capacity of other organelles, such as lysosomes and the nucleus, are small compared to SR and mitochondria. [Ca^2+^]_Cy_ overload is the prerequisite for hypercontracture, whereas [Ca^2+^]_Mi_ is the determinant of MPT. Upon SR Ca^2+^ release, RyR channels situated in close apposition to mitochondria may create a much higher [Ca^2+^] near the outer opening of the mitochondrial Ca^2+^ uniporter channel than the bulk [Ca^2+^]_Cy_, creating a SR-mitochondrial “Ca^2+^ cross-talk” which was shown to significantly contribute to the regulation of [Ca^2+^]_Mi_ [33]. This cross-talk was implicated in facilitating MPT upon reperfusion, so that mitochondrial calcein release, used as an indicator of MPT, was partially blocked by the application of ryanodine/thapsigargin[4]. It was also postulated that upon the event of MPT, mitochondrial Ca^2+^ may redistribute to the cytoplasm, which was implicated in hypercontracture[3] or a regenerative spread of MPT [2].

In our experiments Ni^2+^, the most reliable blocker of NCX, overall blunted [Ca^2+^]_Cy_ dynamics and limited the maximum level of [Ca^2+^]_Cy_ observed during “reperfusion”. This can be considered as the evidence that NCX is a significant source of Ca influx triggering the death sequence in our model. However, there are several arguments against the possibility that reverse-mode NCX operation is the actual mechanism for the massive post-reperfusion increase in [Ca^2+^]_Cy_ observed in our model. First, this event occurred at variable delays after the restoration of normal extracellular milieu. The activation of reverse-mode NCX during reperfusion is linked to the abrupt restoration of the extracellular pH, which triggers influx of Na^+^ through sodium-proton exchanger, which in turn activates reverse-mode NCX extruding Na^+^ and bringing Ca^2+^ in. This sequence of events is expected to occur within the first minute of “reperfusion”. It is not clear why it would occur 15–20 minutes after reintroduction of oxygen and normalization of pH. Second, Zn^2+^ is not transported by NCX and does not influence transport of Ca^2+^ by NCX, and yet in our experiments Zn^2+^ influx was correlated with Ca^2+^ influx, and also interfered with Ca^2+^ influx. Alternative explanation of Ni^2+^ effect may be related to the competitive hinderance by Ni^2+^ of a putative pore which presumably also passes Ca^2+^, Zn^2+^ and TO-PRO3. At the high concentration necessary to block NCX (5 mM) and having the hydrated radius (3.11 Å [34]) smaller than that of Ca2+ (4.1 Å), Ni^2+^ ions could hinder and slow down Ca^2+^ influx through an aqueous pore. In addition, Ni^2+^ intake through the putative pore could have unintended effects inside the cells, possibly contributing to altered dynamics of ΔΨ_m_ and [Ca^2+^]_Mi_ observed in *Nickel* group (compare Figs 3 and 7).

Considering the role of SR, depletion of SR with ryanodine/thapsigargin in our experiments did not affect the sequence of critical events. In fact, depletion of SR accelerated the timing of all critical events (see Fig 5). Speculatively, this could be due to faster cytoplasmic and mitochondrial Ca^2+^ loading in the absence of a major Ca^2+^-absorbing entity.

Lastly, our data does not support the flux of Ca^2+^ from mitochondria to the cytoplasm at any time of reperfusion studied. In all cases, [Ca^2+^]_Mi_ elevation followed [Ca^2+^]_Cy_ elevation at some delay, and after MPT (as indicated by a precipitous loss of ΔΨ_m_) [Ca^2+^]_Mi_ stayed constant or further increased, with the exception of occasional fluctuations (see Fig 3). Moreover, after the onset of the “terminal” [Ca^2+^]_Cy_ rise, abrupt decreases and increases in extracellular [Ca^2+^] immediately propagated to the cytoplasm and mitochondria, symmetrically in inward and outward directions (see Fig 6). Overall, our data suggest that the transition to cell death starts with an abnormal elevation of [Ca^2+^]_Cy_ which occurs via a non-canonical transmembrane conduit, but not due to SR release, and requires some hidden intracellular events to take place after the onset of reperfusion. [Ca^2+^]_Mi_ elevation follows [Ca^2+^]_Cy_ elevation after a ~5 min delay, which probably reflects a limited ability of mitochondria to actively resist Ca^2+^ overload (possibly due to Ca^2+^ buffering by intra-mitochondrial polyphosphates [35]). However, after this ability is saturated or broken, Ca^2+^ ions appear to move freely between extracellular space, cytoplasm, and mitochondrial matrix.

### Sarcolemmal pore expanding during reperfusion—A hypothesis

Whereas for a number of years the focus has been on MPT as the pivotal event in myocardial I/R injury, our findings suggest that MPT is an epi-phenomenon, and an unavoidable consequence, of a critical increase in [Ca^2+^]_Cy._ Since treatment attempting to prevent MPT has not been effective [8], prevention of the [Ca^2+^]_Cy_ elevation may be an effective strategy to salvage cardiomyocytes in the wake of I/R insult, provided we understand the mechanism of this elevation. We believe our data suggests that the critical Ca^2+^ influx occurs through a non-selective sarcolemmal pore expanding with time. A revealing observation here is the relationship between uptake of Ca^2+^, Zn^2+^, and TO-PRO3. The onset of detectable uptake of these species correlated with their estimated size when dissolved in water. The hydrated radii of Ca^2+^ and Zn^2+^ are 4.1 and 4.3 Å, respectively. Stokes radius of TO-PRO3 was not published but the estimated stokes radius of YO-PRO1, a close analog of TO-PRO3, is ~5 Å [36], and we determined in special experiments that TO-PRO3 and YO-PRO1 uptake during “reperfusion” is virtually simultaneous (S5 Fig). It cannot be excluded that the influx of the three species occurred through different channels; but then it would be hard to explain coordination of the presumably independent channels in time. More importantly, there appeared to be specific interactions between the influxes of the three species tested. First, the presence of Zn^2+^ changed the dynamics of Ca^2+^ influx in 57% of analyzed myocytes, introducing a “stairstep” or a “gap” between two phases of [Ca^2+^]_Cy_ rise (see Fig 8). Within the same NRVM monolayer exposed to Zn^2+^ in the perfusate, different cells exhibited variable durations and shapes of these Ca^2+^ influx discontinuities, but those were never observed in *Control* experiments. Also note that in some cells exhibiting the stairstep, the second phase of Ca^2+^ uptake was correlated with the onset of Zn^2+^ uptake (e.g., Fig 7B and 7C). While there isn’t direct evidence as to why this occurred, speculation could envision that Zn^2+^ might have the ability to briefly interfere with the developing pores and to hinder passage of Ca^2+^, if the pores were expanding slowly and were not yet large enough for Zn^2+^ to pass through. As the pores continue to expand, there could be a specific time point when both Ca^2+^ and Zn^2+^ could enter the cell. In cases where this step does not occur, it’s possible that the pores developed more quickly, such that there wasn’t a detectable window of opportunity for Zn^2+^ interference before it could flow into the cell. Lastly, it is worth mentioning that in some cells there was a remarkably similar profile of Zn^2+^ uptake and TO-PRO3 uptake (e.g., Fig 6C). It seems to be improbable that such cases were due to coincidence. Taken together, these different observations could be explained by variations in the dynamics of the pore expansion, which could occur in three stages. If the pore expands really quickly, then the uptake of all three species is very tightly coupled (an indeed we have examples of such cases, see S6 Fig). Otherwise, the relative timing of detectable Ca2+, Zn^2+^, and TO-PRO3 uptake could reflect the time spent by the pore in different stages of expansion.

The expanding pore hypothesis could reconcile discrepancies between findings of Davidson et al. [9], our own previous study [7], and the results of the current study. Amid the pore expansion, the moment of MPT would be determined by the time when the amount of Ca^2+^ absorbed by mitochondria exceeds the threshold for MPT. Depending on the interplay between the pore dynamics and the Ca^2+^ threshold for MPT in different models, the “canonical” SP event detected by uptake of small fluorophores could occur either before [7] or after [9] detectable MPT. Using a genetically encoded cytoplasmic Ca^2+^ indicator in mouse hearts, Davidson et al. detected peculiar Ca^2+^ “waves” which predicted MPT in the same cells. They could not explain the nature of those Ca^2+^ waves. We speculate that the Ca^2+^ waves they saw are equivalent to the critical [Ca^2+^]_Cy_ increases observed in the current study, and may be a result of opening of non-selective pores in the sarcolemma. We also predict that testing the uptake of Zn^2+^ in the whole heart I/R model would show that Zn^2+^ uptake events spatially correlate with Ca^2+^ “waves” and possibly precedes the event of MPT. However, these predictions need to be confirmed in future studies.

Whereas NCX operating in reverse-mode does not seem a be a plausible substrate of the putative expanding pore, there are a few membrane channels that can provide large sarcolemma permeability, and that can become conductive after metabolic inhibition (ischemia/reperfusion). These channels can be formed by proteins identified as connexins or pannexins.

Connexin channels are known to be present at the membrane of many cell types, including cardiac myocytes, and forming structures known as hemichannels [37]. These hemichannels are highly unselective in size and charge [38] and could allow the fast permeation of molecules close to 1KDa, especially during dephosphorylation [39]. Ventricular myocytes express mostly connexin43 (Cx43) [40–42]. Importantly, Cx43 is a phosphoprotein [43], and the conductive properties of the channels they form can be increased during dephosphorylation [44]. Cx43 hemichannels have been shown to open during metabolic inhibition in astrocytes [45, 46] [47]. There is evidence that blockade of Cx43 hemichannels by selective peptide inhibitors is protective in myocardial I/R [48], although the effect is modest [49]. In our experiments requiring continuous perfusion of the imaged cells, the use of Cx43 hemichannels blockers such as gap19 or gap26 was cost-prohibitive; thus, the possibility that Cx43 hemichannels underlie the putative expanding pore remains open. We are working on a miniaturized cell perfusion system which would enable to radically reduce the volume of perfusate and enable application of “gapXX” blockers in our continuous imaging protocol.

Pannexin channels are as complex as connexins; they form unopposed channels in the sarcolemma [50] These channels have been shown also to be highly conductive allowing passage of large molecules, including YO-PR01 [51], and become open during specific metabolic events [52]. They are found associated with ATP-gated ion channels known as P2X receptors which permits an additional conductance in the membrane [53]. A recent study provided evidence that pannexins are expressed in cardiac tissue and that their opening is induced by *Trypanosoma cruzi* infection, which mediate Chagas disease [54]. However, that effect, observed in NRVMs similar to those used in our study, was blocked by 400 μM probenecid. Since in our experiments probenecid was present in all solutions at the concentration of 500 μM, the role of pannexin channels can be ruled out.

### Conclusions

In this study, we established a methodological framework which enabled us to reveal a consistent chronology of critical cellular events during simulated I/R in NRVMs. Despite a highly complex and multi-facetted mechanistic concept of myocardial I/R injury, in our NRVM model cell death upon reperfusion seemed to follow a quite specific path which begins with a pathological [Ca^2+^]_Cy_ increase due to Ca^2+^ influx through a possibly expanding non-selective transmembrane pore. Once a sufficient [Ca^2+^]_Cy_ increase occurred, the subsequent [Ca^2+^]_Mi_ increase followed by MPT are logical and probably unavoidable consequences. The nature of the putative pore remains unknown, but the fact that the abnormal Ca^2+^ uptake route can be hindered by extracellular Zn^2+^, and that Ca^2+^, Zn^2+^, and TO-PRO3 may pass through the same route, should provide initial guidance for steps towards establishing the molecular identity of the putative pore/channel. In future studies it would be important to reproduce the chronology of critical cellular events observed here in a whole heart model of I/R. If the sequence is confirmed, then the putative sarcolemmal pore mediating the abnormal Ca^2+^ influx during reperfusion has to be identified and explored as a target for therapies aimed at salvaging myocardium in the wake of an I/R episode.

## Supporting information

S1 Fig A representative example demonstrating simultaneous cellular loss of Fluo-4 and uptake of TO-PRO3 by an NRVM cell during “reperfusion”. (PDF)

S2 Fig An example of a cell-averaged fluorescence recorded from a NRVM in a sham experiment. Note a relatively constant level of LAR-GECO1.2 and MitoView633 fluorescence, and a continuous presence of Ca transients throughout 110 minutes of continuous recordings in NRVMs perfused with normal oxygenated solution (recordings started 10 min after placing the NRVM monolayer in the perfusion chamber). There was a trend for a slow increase in LAR-GECO1.2 and MitoView633 signal perhaps reflecting slow mitochondrial Ca loading and energizing in beating cells, and a slow decrease in Fluo-4 signal perhaps reflecting an expected slow leak of the dye from the cell. However, there were no loss of MitoView633, or a step-wise increase in LAR-GECO1.2, or a catastrophic increase followed by full dissipation of Fluo-4 signal, as observed in NRVMs subjected to simulated I/R (see Fig 3).
(PDF)

S3 Fig Changes in LAR-GECO1.2 fluorescence in live NRVM cells a function of extracellular pH. Cells were perfused with normal HEPES solution in which pH was varied between 6 and 8. Data presented as mean ± standard deviation (n = 6, in each cell data normalized to the value obtained at pH = 8.0).
(PDF)

S4 Fig Cyclosporine A does not change the sequence of critical events during “reperfusion”. **A**, fluorescence intensity curves during “reperfusion” from a representative cell in the CsA group. All labeling and notations are the same as in Fig 5. **B**, when quantified, the sequence of critical events in the CsA group (grey bars) is the same as in Control group (black bars). *, p < 0.05 as compared to the timing of previous event in the same group (paired t-test); §, p < 0.05 as compared to the timing of the same event in different group (unpaired t-test). In CsA group, T_CaMi_ (the onset of mitochondrial Ca overload) is significantly earlier than in Control group by t-test, but this cannot be explained by the effects of CsA with respect to the MPT pore, and the scientific meaning of this observation remains unclear.
(PDF)

S5 Fig A representative example demonstrating simultaneous cellular uptake of YO-PRO1 and TO-PRO3 by an NRVM cell during “reperfusion”. (PDF)

S6 Fig An example of a NRVM cell in which all critical events occurred almost simultaneously. The time course of fluorescence from 4 different indicators during simulated I/R in a single NRVM, as labeled in the Figure. This cell was selected as an infrequent case where all the critical events were very tightly coupled (2-min time window indicated by light blue), reminiscent of our findings previously published (Ref. 7 in the manuscript). Note that the fluorescence of MitoView633 and TO-PRO3 was recorded in the same channel. However, when segmented separately for the nucleus (grey) and the cytoplasm (red), it was evident that the signal from nucleus, dominated by nucleic acid stain TO-PRO3, started to rise perhaps a minute before the sharp decrease in the cytoplasmic signal, dominated by MitoView633 and reflecting ΔΨ_m_. Also, the uptake of Ca^2+^, Zn^2+^, and TO-PRO3 occur within a minute of each other. We interpret it as the case when the expansion of a putative sarcolemmal pore occurred very quickly, leading to an immediate catastrophe.
(PDF)

S1 Dataset Cell-averaged data for individual *Control* cells. (XLSX)

S2 Dataset Cell-averaged data for individual *Rhya-Thap* cells. (XLSX)

S3 Dataset Cell-averaged data for individual *Nickel* cells. (XLSX)

S4 Dataset Cell-averaged data for individual *CsA* cells. (XLSX)

S5 Dataset Cell-averaged data for individual *Zinc* cells. (XLSX)
