# Supplementary figures and images for: Chronology of critical events in neonatal rat ventricular myocytes occurring during reperfusion after simulated ischemia
Source: PLoS One. 2019 Feb 7;14(2):e0212076. doi: 10.1371/journal.pone.0212076 (PMC6366697; doi:10.1371/journal.pone.0212076)

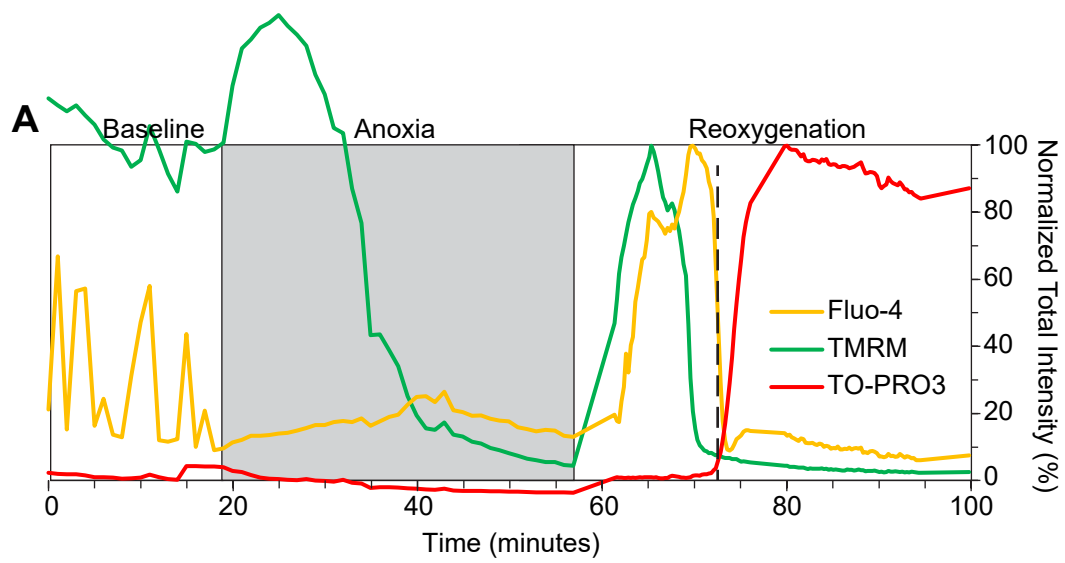

Supplement: S1 Fig — (PDF) [file pone.0212076.s001.pdf]

**A**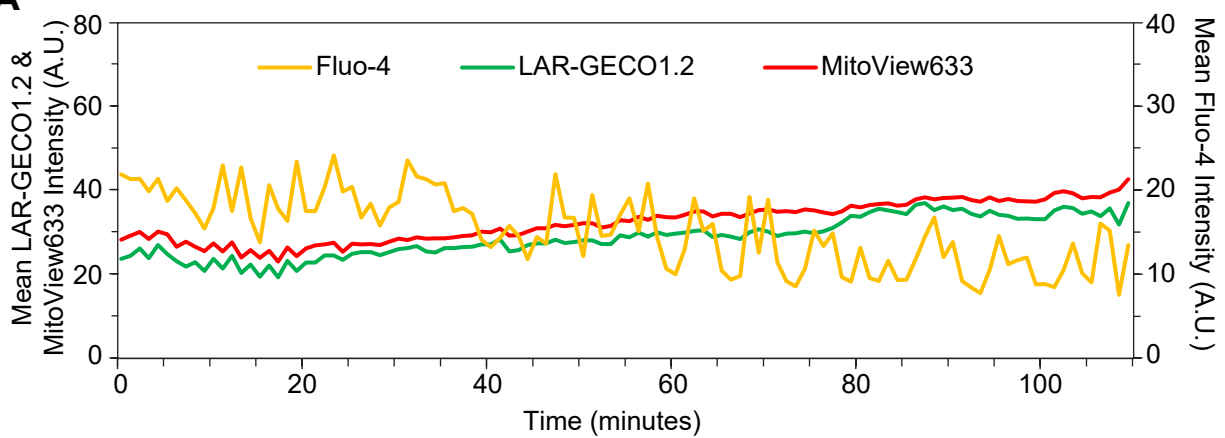

Supplement: S2 Fig — Note a relatively constant level of LAR-GECO1.2 and MitoView633 fluorescence, and a continuous presence of Ca transients throughout 110 minutes of continuous recordings in NRVMs perfused with normal oxygenated solution (recordings started 10 min after placing the NRVM monolayer in the perfusion chamber). There was a trend for a slow increase in LAR-GECO1.2 and MitoView633 signal perhaps reflecting slow mitochondrial Ca loading and energizing in beating cells, and a slow decrease in Fluo-4 signal perhaps reflecting an expected slow leak of the dye from the cell. However, there were no loss of MitoView633, or a step-wise increase in LAR-GECO1.2, or a catastrophic increase followed by full dissipation of Fluo-4 signal, as observed in NRVMs subjected to simulated I/R (see Fig 3). (PDF) [file pone.0212076.s002.pdf]

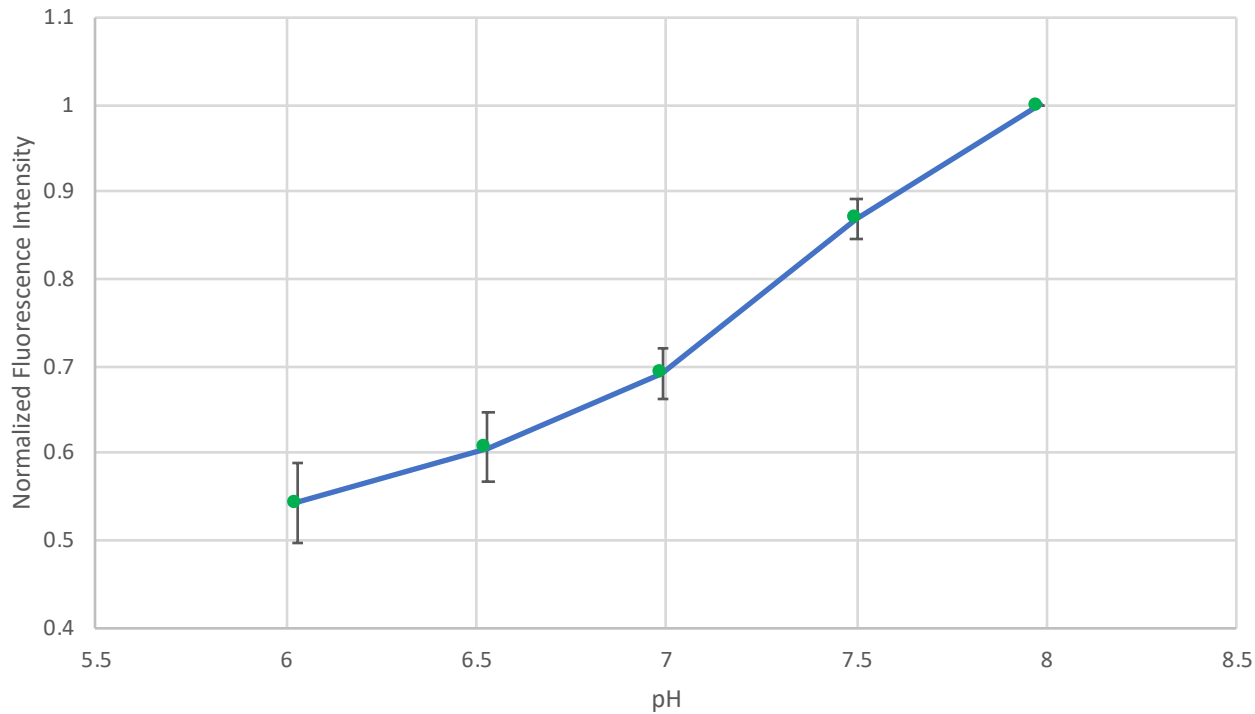

Supplement: S3 Fig — Cells were perfused with normal HEPES solution in which pH was varied between 6 and 8. Data presented as mean ± standard deviation (n = 6, in each cell data normalized to the value obtained at pH = 8.0). (PDF) [file pone.0212076.s003.pdf]

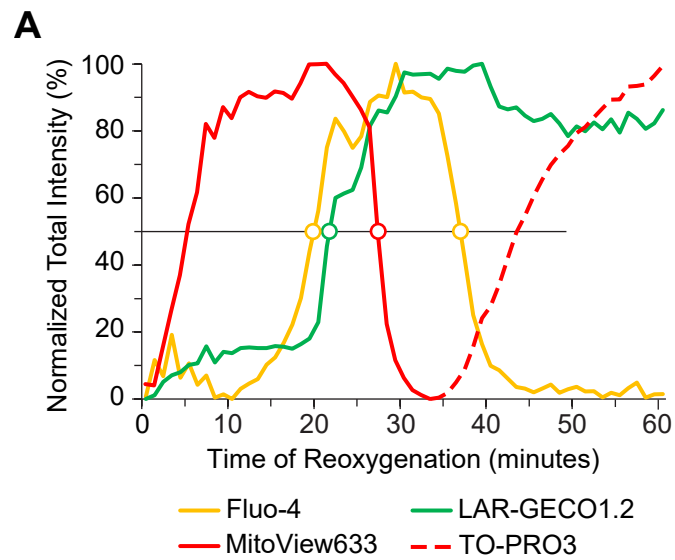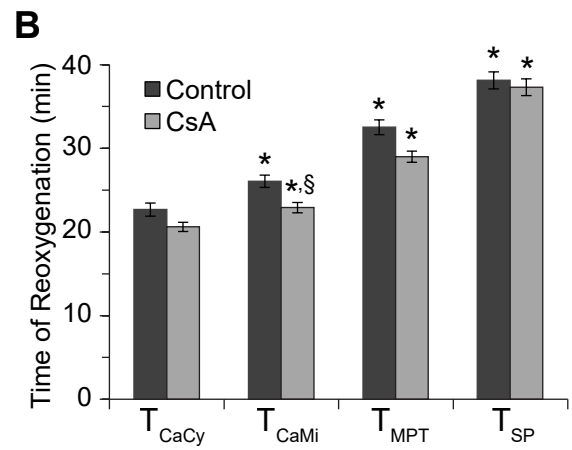

Supplement: S4 Fig — A, fluorescence intensity curves during “reperfusion” from a representative cell in the CsA group. All labeling and notations are the same as in Fig 5. B, when quantified, the sequence of critical events in the CsA group (grey bars) is the same as in Control group (black bars). *, p < 0.05 as compared to the timing of previous event in the same group (paired t-test); §, p < 0.05 as compared to the timing of the same event in different group (unpaired t-test). In CsA group, TCaMi (the onset of mitochondrial Ca overload) is significantly earlier than in Control group by t-test, but this cannot be explained by the effects of CsA with respect to the MPT pore, and the scientific meaning of this observation remains unclear. (PDF) [file pone.0212076.s004.pdf]

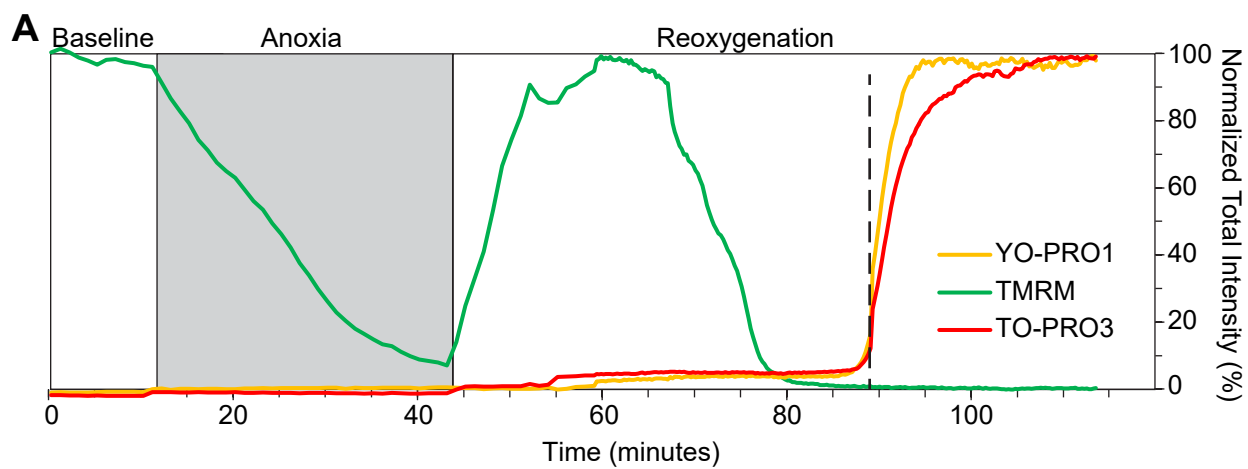

Supplement: S5 Fig — (PDF) [file pone.0212076.s005.pdf]

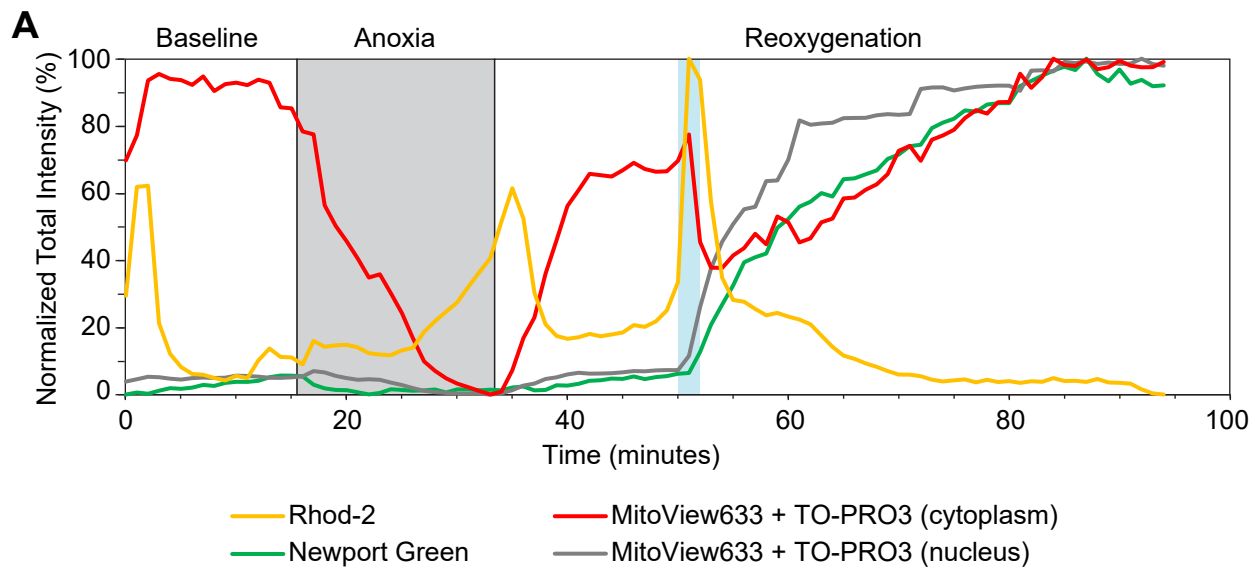

Supplement: S6 Fig — The time course of fluorescence from 4 different indicators during simulated I/R in a single NRVM, as labeled in the Figure. This cell was selected as an infrequent case where all the critical events were very tightly coupled (2-min time window indicated by light blue), reminiscent of our findings previously published (Ref. 7 in the manuscript). Note that the fluorescence of MitoView633 and TO-PRO3 was recorded in the same channel. However, when segmented separately for the nucleus (grey) and the cytoplasm (red), it was evident that the signal from nucleus, dominated by nucleic acid stain TO-PRO3, started to rise perhaps a minute before the sharp decrease in the cytoplasmic signal, dominated by MitoView633 and reflecting ΔΨm. Also, the uptake of Ca2+, Zn2+, and TO-PRO3 occur within a minute of each other. We interpret it as the case when the expansion of a putative sarcolemmal pore occurred very quickly, leading to an immediate catastrophe. (PDF) [file pone.0212076.s006.pdf]
